# Web-Based Training for Nurses on Using a Decision Aid to Support Shared Decision-making About Prenatal Screening: Parallel Controlled Trial

**DOI:** 10.2196/31380

**Published:** 2022-01-25

**Authors:** Alex Poulin Herron, Titilayo Tatiana Agbadje, Sabrina Guay-Bélanger, Gérard Ngueta, Geneviève Roch, François Rousseau, France Légaré

**Affiliations:** 1 VITAM - Research Center on Sustainable Health Centre intégré universitaire de santé et de services sociaux de la Capitale-Nationale Quebec City, QC Canada; 2 Faculty of Nursing University of Laval Quebec City, QC Canada; 3 Department of Epidemiology Faculty of Medicine University of Laval Quebec City, QC Canada; 4 Population Health and Optimal Health Practices Centre Hospitalier Universitaire de Québec - Université Laval Research Centre Quebec City, QC Canada; 5 Centre intégré de santé et de services sociaux de Chaudière-Appalaches Research Center Lévis, QC Canada; 6 Department of Molecular Biology, Medical Biochemistry and Pathology University of Laval Quebec City, QC Canada; 7 Department of Family Medicine and Emergency Medicine Faculty of Medicine University of Laval Quebec City, QC Canada

**Keywords:** shared decision-making, prenatal screening, training, nurses, nursing, behavioral intention, trisomy, Down syndrome, continuing professional development, continuing education, medical education, decision aid, screening, prenatal, pediatrics

## Abstract

**Background:**

Nurses play an important role in supporting pregnant women making decisions about prenatal screening for Down syndrome. We developed a web-based shared decision-making (SDM) training program for health professionals focusing on Down syndrome screening decisions.

**Objective:**

In this study, we aim to assess the impact of an SDM training program on nurses’ intention to use a decision aid with pregnant women deciding on prenatal screening for Down syndrome.

**Methods:**

In this 2-arm, parallel controlled trial, French-speaking nurses working with pregnant women in the province of Quebec were recruited by a private survey firm. They were allocated by convenience either to the intervention group (web-based SDM course that included prenatal screening) or to the control group (web-based course focusing on prenatal screening alone, with no SDM content). The primary outcome was the intention to use a decision aid. Secondary outcomes were psychosocial variables of intention, knowledge, satisfaction, acceptability, perceived usefulness, and reaction to the pedagogical approach. All outcomes were self-assessed through web-based questionnaires, including the space for written comments. We used 2-tailed Student *t* test and Fisher exact test to compare continuous and categorical variables between groups, respectively.

**Results:**

Of the 57 participants assessed for eligibility, 40 (70%) were allocated to the intervention (n=20) or control group (n=20) and 36 (n=18 in each) completed the courses. The mean age of the participants was 41 (SD 9) years. Most were women (39/40, 98%), White (38/40, 95%), clinical nurses (28/40, 70%), and had completed at least a bachelor’s degree (30/40, 75%). After the intervention, the mean score of intention was 6.3 (SD 0.8; 95% CI 5.9-6.7) for the intervention group and 6.0 (SD 1.2; 95% CI 5.42-6.64) for the control group (scale 1-7). The differences in intention and other psychosocial variable scores between the groups were not statistically significant. Knowledge scores for SDM were significantly higher in the intervention group (79%, 95% CI 70-89 vs 64%, 95% CI 57-71; *P*=.009). The intervention was significantly more acceptable in the intervention group (4.6, 95% CI 4.4-4.8 vs 4.3, 95% CI 4.1-4.5; *P*=.02), and reaction to the pedagogical approach was also significantly more positive in the intervention group (4.7, 95% CI 4.5-4.8 vs 4.4, 95% CI 4.2-4.5; *P*=.02). There was no significant difference in overall satisfaction (or in perceived usefulness). Furthermore, 17 participants (9 in the intervention group and 8 in the control group) provided written comments on the intervention.

**Conclusions:**

This study focuses on web-based nursing education and its potential to support pregnant women’s decision-making needs. It shows that nurses’ intention to use a decision aid to enhance SDM in prenatal care is high, with or without training, but that their knowledge about SDM can be improved with web-based training.

**International Registered Report Identifier (IRRID):**

RR2-10.2196/17878

## Introduction

### Background

In Quebec, prenatal screening for trisomy 21 (Down syndrome) during prenatal follow-up is offered to all pregnant women as part of the state-run health care services, as well as in a few private clinics [1]. Pregnant women face several possible options: whether to take the test, and if so, which one (biochemical test, fetal DNA, or nuchal translucency ultrasound) [2,3]. Prenatal screening is a value-laden decision, and the probabilistic nature of the evidence makes it a difficult decision for expecting parents. It is also complex because new difficult decisions may arise if the results of screening indicate a high probability of Down syndrome in the fetus—that is, whether to perform a more invasive procedure to confirm or deny the screening results (which includes risks of miscarriage) and, ultimately, whether to continue or terminate the pregnancy, given that prenatal treatment options are not available [4].

Faced with the complex nature of this decision, future parents must not only be informed of the evidence regarding the tests but also be supported by the health care team so that their preferences and values are considered in the decision-making process [5]. Thus, shared decision-making (SDM) seems to be the most appropriate approach for setting out the possible options in the most objective and concrete way, fostering informed consent and empowering parents [6-8]. SDM improves the health care experiences of both patients and health professionals and leads to better health care processes, patient experience and outcomes, and optimal health spending [9-12]. To implement SDM in clinical practice, several approaches have been proposed in the literature, including interactive skills workshops for health professionals and implementation of SDM tools known as decision aids (DAs) [13,14]. DAs are tools used by health care professionals to assist patients in their decision-making by providing information on treatment or screening options and their associated benefits and harms and by clarifying their values and preferences regarding the decision to be made [15].

In Canada, prenatal care requires the collaboration and coordination of many health care providers [16,17]. Nurses play an essential role, especially in the province of Quebec. One of their roles is to initiate laboratory examinations and diagnostic tests, such as prenatal screening [18]. Thus, they are in a strategic position to implement SDM as they accompany pregnant women and their families in decision-making about prenatal screening [18,19]. Owing to their close and trusting relationship with patients, their intimate understanding of their community environment, their understanding of biology, and their communication skills, nurses could be powerful allies in implementing SDM in prenatal care [20]. Most SDM implementation studies focus on physicians [21], but with the transformation of health care systems, nurses are increasingly involved in clinical decision-making, and their crucial role urgently needs acknowledgment [22]. Patients themselves have expressed that nurses could play a significant role in SDM; they could provide information and support as well as communicate with other clinicians [23]. Moreover, many studies highlight that an interprofessional approach to SDM, in the context of team-based health care, has benefits for SDM implementation [24-28]. Investigating nursing training in SDM not only gives us an underexplored perspective but could also inform us about the complexities involved in interprofessional SDM.

However, for nurses to implement SDM in prenatal care, they must acquire the skills to apply this approach in their practice. Web-based continuing professional development (CPD) increased 10-fold from 2002 to 2008 in the United States and has been accelerating ever since [29,30]. The continuing education needs of remote rural practices and, more recently, COVID-19 social distancing rules and health cuts have driven developers of CPD to provide more web-based courses [31,32]. Although the effect of web-based courses on clinical practice is still not clear [33], some evaluations show that they are just as effective as in-person courses [34].

### Objective

Therefore, we seek to assess the impact of a web-based SDM training program on nurses’ intention to use a DA with pregnant women facing a decision about prenatal screening for Down syndrome.

## Methods

### Overview

We used the CONSORT-EHEALTH (Consolidated Standards of Reporting Trials of Electronic and Mobile Health Applications and Online Telehealth) checklist (version 1.6.1) as a reporting guideline (except for items related to randomization) to report this study (Multimedia Appendix 1). This checklist suggests information to include when reporting eHealth or mobile health trials (not only web-based or internet-based interventions and DAs but also social media, serious games, DVDs, mobile apps, and certain telehealth applications) [35]. We also used the guidelines for reporting nonrandomized pilot and feasibility studies proposed by Lancaster and Thabane [36].

### Study Design

This study was a 2-arm, parallel controlled trial with pre–post measures. Participants were conveniently allocated to two parallel groups: (1) an intervention group exposed to a 3-hour web-based course on SDM, including SDM for prenatal screening, or (2) a control group exposed to a 3-hour web-based course on prenatal screening alone with no SDM content.

### Changes to Methods

Initially, this study was planned as a randomized controlled trial. Through a misunderstanding, however, the private firm who performed the recruitment used convenience allocation, and there was no randomization allocation. No content changes were made to the interventions, and no software malfunctions occurred during the interventions.

### Research Approval

This project was approved by the ethics committee of the Centre Hospitalier Universitaire de Québec-Université Laval (MP-20-2019-4571). All stages of this research project were conducted in accordance with the rules of ethics. Further details on ethics can be found in the protocol by Poulin Herron et al [37].

### Study Population

Inclusion criteria for nurses were that they (1) were involved in prenatal screening decision-making or prenatal screening processes in the province of Quebec; (2) spoke and wrote French; (3) were active in professional practice during the year of data collection (eg, hospitals and community clinics); and (4) had sufficient internet skills, as all procedures were web-based, requiring a minimum of ability and equipment to enter and navigate through the course. There were no exclusion criteria.

### Procedures and Recruitment

Participants were recruited through a web-based approach by a private polling firm that operates an internet panel. Recruitment made use of (1) a pre-existing list of nurses held by the polling firm; (2) social media; and (3) the human resources departments of two regional health authorities, the Centre intégré universitaire de santé et de services sociaux de la Capitale-Nationale and the Centre intégré de santé et de services sociaux de Chaudière-Appalaches. Recruitment and enrollment took place on web. The procedures are described in detail in a previously published protocol [37].

### Allocation

The allocation of participants to the trial groups was performed after collecting the sociodemographic data. The private firm performed the allocation by convenience, as the nurses entered the study. Participants were not blinded throughout the study but were also not told which arm (intervention or control) they were allocated.

### Study Interventions

#### Overview

The SDM course was created by members and collaborators of the Canada Research Chair in Shared Decision Making and Knowledge Translation with help from education counselors from Université Laval and supported by the PEGASUS (Personalized Genomics for Prenatal Abnormalities Screening Using Maternal Blood) program, a pan-Canadian program. Nurse with expertise in this field created a prenatal screening course without SDM content. The mode of delivery was asynchronous and web-based.

Both programs, hosted on the Université Laval platform, could be completed in approximately 3 hours. More information about the programs can be found in the protocol by Poulin Herron et al [37]. Some participants required technical assistance with regard to the study questions (eg, about when the course is considered complete or when they should answer the postintervention questionnaire), but no technical problems arose related to the course or the platform. Multimedia Appendix 2 provides a report of both programs according to the TIDieR (Template for Intervention Description and Replication items) [38].

#### Intervention Group: Web-Based Course on SDM and Down Syndrome Prenatal Screening

The intervention consisted of a web-based self-study course titled *Formation en ligne – La prise de décision partagée pour le test de dépistage prénatal de la trisomie 21* (Shared Decision-Making About Prenatal Screening for Down Syndrome). The course aimed to integrate SDM into prenatal care by focusing on the use of a DA. It was divided into four main modules: (1) SDM, (2) Down syndrome prenatal screening, (3) DAs, and (4) communication between health care professionals and patients. Course development was based on the SDM model [39] and its central concepts [14,39]. These include assessment with the patient and health care professional that there is a decision to be made, exploration of the options available and their pros and cons, discussion about patients’ preferences, support of the patient by health care professionals in their decision-making, and discussion surrounding the decision to be made. The course depicted a DA and included a video of a clinical encounter in which the DA was used by a clinician with expecting parents. The DA was created by our research team [40] using the International Patient Decision Aids Standards [41]. The model used in the course was in paper format. Its core element is the decision of whether to undergo prenatal screening for Down syndrome. It describes Down syndrome; presents the various prenatal tests and their benefits, harms, and consequences; and provides an exercise for users to clarify their values regarding the options. Multimedia Appendix 3 includes screenshots of the training content.

#### Control Group: Web-Based Course on Down Syndrome Prenatal Screening

The control group course was titled *Formation sur le dépistage prénatal de la trisomie 21* (prenatal screening for Down syndrome) and focused on prenatal screening alone (without SDM content such as determining decision points or using a DA). In the control program, the topic of SDM in module 1 of the intervention arm was replaced with “Context and history of prenatal screening.” The topic of DAs in module 3 of the intervention arm was replaced with “Consent in prenatal screening.” There were no DA and SDM simulation videos.

### Data Collection

For each study arm, data were collected before and after the nurses completed the courses. All outcomes were self-reported. No postintervention data were collected from the participants who discontinued the intervention. The Kirkpatrick and Kirkpatrick [42] model was used as an overall guide to evaluate the effectiveness of the course, as it has proven useful in guiding the evaluation of training for the health care provider [43]. This model comprises four levels: (1) reaction, (2) learning, (3) behavioral change, and (4) organizational performance (results). Level 1 (reaction) measures how participants react to training (eg, satisfaction). Level 2 (learning) analyzes whether they understood the training (eg, increase in knowledge). Level 3 (behavior) looks at whether they are using what they learned at work (eg, behavior change), and level 4 (results) determines whether the material has a positive impact on the organization [42]. Details on the rationale for the questionnaire guides (Multimedia Appendix 4 summarizes the data collection questionnaire after the training) and further explanations can be found in the protocol by Poulin Herron et al [37]. Data collection took place entirely on web. Participants received CAD $400 (US $315) for their participation.

### Primary Outcome

The primary outcome was the intention of nurses to use a DA in clinical practice with pregnant women facing a decision about prenatal screening for Down syndrome. Intention was chosen as the primary outcome because of the literature supporting intention as a strong predictor of behavior [44-46]. Intention is defined as the degree to which a person has formulated conscious plans to perform or not perform some specified future behavior, that is, the subjective probability that they will perform the behavior [47]. According to the integrated model of the theory of planned behavior for health care providers by Godin [46], their intention to change their behavior can be predicted by four variables: social influence, beliefs about capabilities, moral norms, and beliefs about consequences, as well as by individual and environmental characteristics. The intention to use a DA could act as a proxy for level 3 in the Kirkpatrick and Kirkpatrick [42] model, that is, a proxy for changes in behavior. Thus, although nurses’ mid- or long-term behavior was not evaluated in this study, we assumed that their intention to use a DA in clinical practice with pregnant women could predict their future SDM behavior in clinical practice after receiving the training and once the DA is implemented in clinical settings. Further details on the rationale for the choice of primary outcome can be found in the protocol by Poulin Herron et al [37].

Intention was measured before the intervention (before the course) and after the intervention (within 24-72 hours after completing the course). It was assessed using the CPD-Reaction questionnaire [48], which measures intention (and its psychosocial variables) to change a behavior after completing a CPD activity. The tool is based on the theory of planned behavior [46,49] and Triandis theory [50]. This validated questionnaire has an acceptable internal consistency for each construct, with a Cronbach *α* ranging from .77 to .85 [45]. Intention (questions 1 and 7 of the questionnaire) was measured on a 7-point Likert scale ranging from 1 (strongly disagree) to 7 (strongly agree).

### Secondary Outcomes

Secondary outcomes included the psychosocial variables of behavioral intention as evaluated with the CPD-Reaction questionnaire, that is, social influence, beliefs about capabilities, moral norms, and beliefs about consequences. These constructs were also measured on a 7-point Likert scale, from 1 (score indicating a weak determinant) to 7 (score indicating a strong determinant). Question 2, on social influence, or the percentage of one’s colleagues who use DAs, is on a 5-point Likert scale from 1 (0%-20%) to 5 (81%-100%) [48].

Knowledge (level 2 in the Kirkpatrick and Kirkpatrick [42] model) was measured using a 20-item questionnaire designed by the research team and focused on 4 topics: Down syndrome (2 items), prenatal screening (7 items), SDM (7 items), and ethics (4 items). The questions had from 2 to 5 possible answers, with both true-or-false–type answers and multiple-choice answers. As the knowledge questionnaire did not use Likert scales, the knowledge score was evaluated as a percentage with a maximum knowledge score of 100%.

To measure nurses’ overall impression (level 1 in the Kirkpatrick and Kirkpatrick [42] model) of the web-based course in which they were enrolled, we assessed their satisfaction, acceptability, and perceived usefulness of the course, and reaction to its pedagogical approach. We used the questionnaire by Körner et al [26] to assess satisfaction with the content (5 items), with the trainers (6 items), and overall satisfaction. We used the Giangreco [51] questionnaire to assess the perceived usefulness of the program. We assessed acceptability using a 5-item questionnaire based on the Kirkpatrick and Kirkpatrick [42] guidelines for evaluating reactions to educational programs. Finally, we assessed the reaction to the pedagogical approach using a 9-item questionnaire based on both the Kirkpatrick and Kirkpatrick [42] guidelines and the University of Connecticut School of Medicine’s continuing medical education evaluation form [52]. All 4 level 1 variables were assessed using a 5-point Likert scale ranging from 1 (strongly disagree) to 5 (strongly agree).

In open fields, we also collected written comments on how to improve training and sociodemographic data. No changes to trial outcomes were made after the trial commenced.

### Data Management

All data collected were kept on the polling firm’s secure server for 10 years. Following data collection, the firm sent a deidentified database of all data collected in a Microsoft Excel file (version 2019) and a SAS (version 9.4) file to the research team. An identification number was given to each participant to deidentify and track them throughout the study. The research team saved these data on the secure server of regional health authorities.

### Sample Size

To detect an average difference in our primary outcome (intention to use a DA), it was estimated that a sample size of 36 nurses (n=18 per group) would be sufficient, with an error of 0.05, a size effect of 0.8, and a power of 80%. This sample size was based on a similar study assessing intention to use a DA for Down syndrome screening among midwives, a profession closely allied with nursing [53].

### Data Analysis

Analyses were performed at the individual level. We used the 2-tailed Student *t* test and Fisher exact test [54] to compare continuous and categorical variables between groups (mean intention to use the DA in both groups, knowledge and overall impressions). The difference in scores was estimated by subtracting the preintervention score from the postintervention score. This pre–post measure of change followed a normal distribution and was used in the regression model as a response variable without any transformation. The exposure factor under study was the intervention (vs the control group). The identification of confounding factors was carried out using the 10% method [55], and variables whose removal from the full model (ie, including exposure factor and all potential covariates) resulted in a change of ≥10% in the effect of the intervention were retained as confounding variables in the final model. To compare the mean values of secondary outcomes between the 2 groups after the intervention, analyses of covariance models were performed to control for confounding factors. For all analyses, SAS was used, and a statistical significance level of .05 was defined. No imputation techniques were used to deal with attrition or missing values, as the sample size of this study could not handle such an analysis. Finally, we conducted a content analysis of written comments. All results are presented as mean scores or percentages with 95% CIs in parentheses.

## Results

### Participant Characteristics

**Figure 1:**
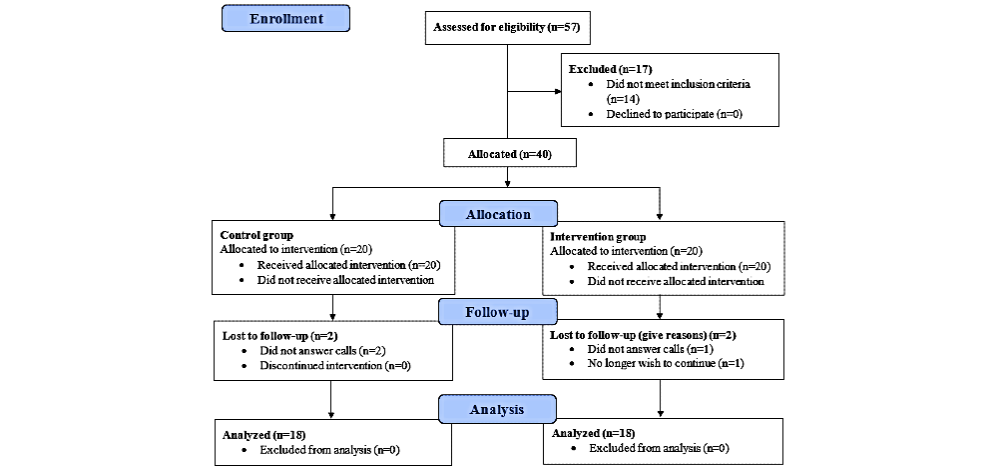
Participant flow diagram.

Data were collected from September 2019 to late January 2020. A total of 57 potentially eligible nurses were assessed for this trial, of whom 40 were allocated to either the control group or the intervention group. Two participants per group (total n=4) discontinued participation in the study (ie, they did not complete the full module). The trial ended when 18 nurses in each group completed the course and the postintervention questionnaire (Figure 1).

### Baseline Data

The mean age of the participants was 41 (SD 9) years. Only one male enrolled, and all participants were cisgender people. Most participants had completed at least a bachelor’s degree (26/40, 65%), resided in urban regions (19/40, 47.5% in the Capitale-Nationale and 9/40, 22.5% in Chaudière-Appalaches), were White (38/40, 95%), earned between CAD $60,000 and CAD $99,999 (US $46,885 and US $78,144) (30/40, 75%), were clinical nurses (university vs college-trained; 28/40, 70%), and worked full-time (35/40, 87.5%) either in a hospital (19/40, 47.5%) or a local community health center (7/40, 35%). The number of pregnant women the nurses had seen in the past month varied: 45% (18/40) had seen from 1 to 20, 15% (6/40) had seen from 21 to 40, 15% (6/40) had seen from 41 to 80, and 18% (7/40) had seen ≥81. Moreover, 3 nurses (7.5%) had not seen any pregnant women in the past month (Table 1). The intervention and control groups did not statistically differ with respect to demographic data.

**Table 1:** Participants’ sociodemographic characteristics.

			Total (n=40)		Intervention (n=20)		Control (n=20)
Age (years), mean (SD)			40.7 (9.4)		39.5 (9.8)		41.8 (9.1)
**Biological sex, n (%)**							
	Male	1 (2.5)		1 (5)		0	
	Female	39 (97.5)		19 (95)		20 (100)	
	Other	0 (0)		0		0	
**Gender, n (%)**							
	Male	1 (2.5)		1 (5)		0	
	Female	39 (97.5)		19 (95)		20 (100)	
	Other	0 (0)		0		0	
**Formal education^a^, n (%)**							
	College (CEGEP)	9 (22.5)		5 (12.5)		4 (20)	
	University—bachelor	26 (65)		14 (70)		12 (60)	
	University—master	4 (10)		0		4 (20)	
	University—doctorate	0 (0)		0		0	
	Other	1 (2.5)		1 (5)		0	
**Residency^a^, n (%)**							
	Capitale-nationale	19 (47.5)		10 (50)		9 (45)	
	Chaudières-Appalaches	9 (22.5)		4 (20)		5 (25)	
	Laval	3 (7.5)		1 (5)		2 (10)	
	Montréal	4 (10)		1 (5)		3 (15)	
	Bas-Saint-Laurent	1 (2.5)		1 (5)		0	
	Abitibi-Téminscamingue	1 (2.5)		1 (5)		0	
	Gaspésie and Îles-de-la-madeleine	1 (2.5)		1 (5)		0	
	Center-du-Québec	1 (2.5)		0		1 (5)	
	Other	1 (2.5)		1 (5)		0	
**Ethnicity^a^, n (%)**							
	White	38 (95)		19 (95)		19 (95)	
	Racialized minority	2 (5)		1 (5)		1 (5)	
**Salary^a^ (CAD $), n (%)**							
	<CAD $29,999 (US $23,444)	1 (2.5)		0		1 (5)	
	CAD $30,000–$59,999 (US $23,445–$46,884)	5 (12.5)		3 (15)		2 (10)	
	CAD $60,000–$99,999 (US $46,885–$78,144)	30 (75)		15 (75)		15 (75)	
	≥CAD $100,000 (US $78,145)	4 (10)		2 (10)		2 (10)	
**Job title^a,b^, n (%)**							
	Nurse	11 (27.5)		8 (40)		3 (15)	
	Clinical nurse	28 (70)		12 (60)		16 (80)	
	Nurse practitioner	1 (2.5)		0		1 (5)	
**Job status^a^, n (%)**							
	Preventive withdrawal or maternity leave	1 (2.5)		0		1 (5)	
	Full-time	35 (87.5)		19 (95.5)		16 (80)	
	Part-time	3 (7.5)		1 (5)		2 (10)	
	Retired	1 (2.5)		0		1 (5)	
**Work settings**							
	Hospital	19 (47.5)		8 (40)		11 (55)	
	Family medicine group	1 (2.5)		0		1 (5)	
	Local community services centers	14 (35)		7 (35)		7 (35)	
	Other	6 (15)		5 (25)		1 (5)	
**Estimated number of pregnant women seen in the last month**							
	0	3 (7.5)		1 (5)		2 (10)	
	1-5	7 (17.5)		6 (30)		1 (5)	
	6-20	11 (27.5)		6 (30)		5 (25)	
	21-40	6 (15)		2 (10)		4 (20)	
	41-80	6 (15)		3 (15)		3 (15)	
	≥81	7 (17.5)		2 (10)		5 (25)	

^a^To simplify the table, we did not present all the subvariables for the sociodemographic variables marked with “a.” Subvariables not presented in the table contained no data. For the *formal education* variable, the subvariables not presented in the table are *none completed, primary school, secondary school*, and *professional study diploma*. One participant marked other on their form, referring to a university certificate. The work setting was not found to be a confounding factor. For the variable *residency*, the subvariables not presented are *Saguenay-Lac-Saint-Jean, Mauricie, Estrie, Outaouais, Côte-Nord, Nord-du-Québec Lanaudières, Montérégie, Laurentides*, and *I do not know*. For the variable *ethnicity*, subvariables not presented are *Latino-American* (Mexico, Chili, Costa Rica, etc), *Arab* (Middle East, Maghreb, etc), *Sud-Asia* (India, Bangladesh, Pakistan, Sri Lanka, etc), *South-east Asia* (Vietnam, Cambodia, Malaysia, Laos, etc), *West Asia* (Iran, Afghanistan, etc), *Chinese, Filipino, Korean, Japanese, Other*, and *I do not know*. For the variable *salary*, subvariables not presented are *I do not know* and *I prefer not to answer*. For the variable *job title*, the subvariable not presented is *none of the above*. For the variable *job status*, the subvariables that are not presented in the table are *student, no job/studies*, and *none of the above*. Other types of work settings referred to private clinics, nursing stations, First Nations services, or federal (as opposed to provincial) services.

^b^The difference between nurses and clinical nurses is their education level, proper to the province of Quebec; clinical nurses have a bachelor’s degree, and nurses have a college degree; both have similar scope of practice, with distinctions in regard to care complexity, coordination and clinical supervision.

### Primary Outcome: Intention

Before the intervention, the mean intention score was 6.2 (SD 0.9; 95% CI 5.8-6.7) for the intervention group and 5.9 (SD 1.4; 95% CI 5.2-6.5) for the control group. The minimum and maximum scores were 1 and 7, respectively. After the intervention, the mean intention score was 6.3 (SD 0.8; 95% CI 5.9-6.7) for the intervention group and 6.0 (SD 1.2; 95% CI 5.4-6.6) for the control group (Table 2). Between the pre- and postintervention stage, the difference in intention score was 0.1 (95% CI −0.5 to 0.6) for the intervention group and 0.1 (95% CI −0.8 to 1.1) for the control group. Before the intervention, the difference in intention score between the intervention and control groups was 0.3 (95% CI −0.4 to 1.1). After the intervention, the difference in intention score between intervention and control groups was 0.3 (95% CI −0.4 to 1.0). The intra- and intergroup differences observed were not statistically significant. After adjustment for confounding variables, the pre–post change in intention scores did not vary significantly among the exposure groups (0.2, 95% CI −1.0 to 1.4; *P*=.74).

**Table 2:** Continuing Professional Development–Reaction construct scores.

		Before intervention				After intervention				*P* value	
		Total (n=40)	Intervention (n=20)	Control (n=20)	Total (n=36)		Intervention (n=18)	Control (n=18)			
**Intention**											.82
	Values, mean (SD; 95% CI)	6.1 (1.2; 5.7-6.4)	6.2 (0.9; 5.8-6.7)	5.9 (1.4; 5.2-6.5)	6.2 (1.0; 5.8-6.5)		6.3 (0.8; 5.9-6.7)	6.0 (1.2; 5.4-6.6)			
	Values, median (IQR)	6.3 (5.5-7.0)	6.5 (5.5-7.0)	6.0 (5.5-7.0)	6.5 (5.8-7.0)		6.5 (6.0-7.0)	6.5 (5.5-7.0)			
**Social influence**											.15
	Values, mean (SD; 95% CI)	3.4 (1.7; 2.9-4.0)	2.7 (1.6; 2-3.4)	3.8 (1.7; 2.9-4.6)	3.0 (1.4; 2.5-3.4)		2.7 (1.3; 2.1-3.4)	3.2 (1.5; 2.4-3.9)			
	Values, median (IQR)	3.1 (1.8-4.8)	2.5 (1.5-3.6)	4.3 (2.7-5.1)	2.5 (1.8-3.8)		2.3 (1.5-3.9)	2.7 (2.5-3.6)			
**Beliefs about capabilities**											.07
	Values, mean (SD; 95% CI)	5.7 (1.0; 5.4-6.1)	6.1 (1.0; 5.7-6.5)	5.4 (1.0; 4.9-5.9)	5.8 (0.8; 5.5-6.1)		5.8 (0.8; 5.5-6.2)	5.8 (0.9; 5.4-6.2)			
	Values, median (IQR)	6.0 (5.0-6.7)	6.3 (5.0-6.8)	5.7 (4.7-6.3)	6.0 (5.3-6.3)		6.0 (5.3-6.3)	6.0 (5.3-6.3)			
**Moral norm**											.34
	Values, mean (SD, 95% CI)	6.6 (0.6; 6.4-6.8)	6.9 (0.4; 6.7-7.0)	6.4 (0.7; 6.0-6.7)	6.5 (0.8; 6.2-6.7)		6.6 (0.9; 6.1-7.0)	6.4 (0.7; 6.0-6.7)			
	Values, median (IQR)	7.0 (6.0-7.0)	7.0 (6.8-7.0)	6.5 (6.0-7.0)	7.0 (6.0-7.0)		7.0 (6.5-7.0)	6.5 (6.0-7.0)			
**Beliefs about consequences**											.68
	Values, mean (SD; 95% CI)	6.5 (0.6; 6.3-6.7)	6.8 (0.5; 6.6-7)	6.3 (0.7; 6-6.6)	6.6 (0.8; 6.4-6.9)		6.9 (0.4; 6.7-7.1)	6.3 (0.9; 5.9-6.8)			
	Values, median (IQR)	7.0 (6.0-7.0)	7.0 (6.5-7.0)	6.0 (6.0-7.0)	7.0 (6.5-7.0)		7.0 (7.0-7.0)	6.8 (6.0-7.0)			

### Secondary Outcomes

#### Other Constructs of the CPD-Reaction Questionnaire

Scores for social influences, beliefs about capabilities, moral norms, and beliefs about consequences are shown in Table 2. There were no statistically significant differences between the pre- and postintervention scores for any of these constructs.

#### Knowledge

Table 3 shows knowledge scores assessed after the intervention. The average score for the 7 knowledge questions about SDM was 79% (SD 18; 95% CI 70%-89%) in the intervention group and 64% (SD 14; 95% CI 57%-71%) in the control group. The mean difference between the 2 groups for knowledge about SDM was 15% (SD 16; 95% CI 4%-26%). This difference is statistically significant (*P*=.009). There was no statistically significant difference in the knowledge scores for the questions about Down syndrome, ethics, or prenatal screening.

**Table 3:** Knowledge scores after the intervention (n=18).

Topics	Intervention (n=18), mean (SD; 95% CI)	Control (n=18), mean (SD; 95% CI)	Mean difference (SD; 95% CI)	*P* value
Shared decision-making (7 items; %)	79 (18; 70 to 89)	64 (14; 57 to 71)	15 (16; 4 to 26)	.009
Down syndrome (2 items; %)	89 (21; 78 to 100)	78 (31; 62 to 93)	11 (27; −7 to 29)	.21
Ethics (7 items; %)	68 (21; 58 to 78)	69 (22; 59 to 80)	−1 (21; −16 to 13)	.85
Prenatal screening (4 items; %)	74 (13; 67 to 80)	79 (15; 71 to 86)	−5 (14; −14 to 5)	.32

#### Participants’ Overall Impression of the Training

Table 4 shows scores of satisfaction (with content, with trainers, and overall satisfaction), acceptability, perceived usefulness, and reaction to the pedagogical approach. Item scores for participants’ impression of the training was based on a scale of 1 to 5. The mean score for acceptability of the training program was 4.6 (95% CI 4.4-4.8) in the intervention group and 4.3 (95% CI 4.1-4.5) in the control group. The difference in acceptability of training between the 2 groups was statistically significant (*P*=.02). The mean score of reaction to the pedagogical approach was 4.7 (95% CI 4.5-4.8) in the intervention group and 4.4 (95% CI 4.2-4.5) in the control group (ie, the intervention group reacted more positively). This difference in reaction between the 2 groups was statistically significant (*P*=.02). The between-group differences in scores of satisfaction and perceived usefulness were not statistically significant.

**Table 4:** Overall impression of the course (scale of 1-5).

			Intervention (n=18), Mean (SD; 95% CI)		Control (n=18), Mean (SD; 95% CI)		Mean difference (SD; 95% CI)		*P* value
**Satisfaction**									
	Content	4.4 (0.9; 3.9 to 4.8)		4.2 (0.4; 4.02 to 4.4)		0.1 (0.7; −0.3 to 0.6)		.53	
	Trainers	4.6 (1.0; 4.1 to 5.02)		4.2 (0.4; 4.1 to 4.4)		0.3 (0.7; −0.2 to 0.8)		.21	
	Overall satisfaction	4.5 (1.0; 4.01 to 4.9)		4.2 (0.4; 4.05 to 4.4)		0.2 (0.7; −0.2 to 0.7)		.31	
Acceptability			4.6 (0.4; 4.4 to 4.8)		4.3 (0.4; 4.1 to 4.5)		0.3 (0.4; 0.1 to 0.6)		.02
Perceived usefulness			4.6 (0.4; 4.4 to 4.8)		4.4 (0.5; 4.2 to 4.6)		0.2 (0.4; −0.1 to 0.5)		.13
Reaction (pedagogical aspects)			4.7 (0.4; 4.5 to 4.8)		4.4 (0.4; 4.2 to 4.5)		0.3 (0.4; 0.04 to 0.5)		.02

### Written Comments

Overall, 17 participants (9 in the intervention group and 8 in the control group) provided feedback on the course. Most participants in the intervention group (6/9, 67%) described the training as excellent, perfect, practical, applicable, or relevant. One participant expressed comfort with the DA and the principle of SDM. One said that she would use the DA as soon as possible. Moreover, 2 participants emphasized the usefulness of the training, which they felt increased their knowledge and confidence and gave them the tools to better structure meetings with their patients:

I was a little uncomfortable bringing up this topic [talking about prenatal screening for Down syndrome]...this part of the information was more to discuss with the doctor...It will allow me to better structure my meetings and I feel much better equipped.

Participant 9

However, 2 nurses pointed out that, in practice, they lack the time to use such tools and that the rapid sequence of steps leading to prenatal screening affords future parents little time for reflection:

I work in a local community health centre [CLSC] with 5 consultations per day, where there’s little time allocated per client. For me it would be difficult to use this decision aid. However, if I had more time, I could, and I would find it practical and very useful.

Participant 25

In the control group, although some participants (5/8, 63%) were happy to have had the opportunity to do this course and found it interesting, 2 of them pointed out the difficulty in understanding the person who was speaking on the PowerPoint narration videos because of their rapid speech, unclear pronunciation, and changes in intonation (control intervention only). In addition, 3 of them said that the length of the first module and the length of documents to be consulted made the course longer than 3 hours.

## Discussion

### Principal Findings

We assessed the impact of an SDM course on nurses’ intention to use a DA with pregnant women facing the decision of prenatal screening for Down syndrome, as well as the nurses’ overall impressions of the SDM training. We found no statistically significant difference in intention scores between the intervention arm (SDM course) and the control arm (course on the screening program only). However, we found a statistically significant difference in knowledge about SDM, acceptability, and reaction to the pedagogical approach between the 2 arms. In written comments, nurses identified lack of time as a barrier to the use of the DA in clinical settings. These results lead us to make the following observations.

First, there was no difference in intention scores between the intervention and control groups. This was a comparative effectiveness study [56], as both arms presented an active intervention. Although the control arm developed by nurse experts in prenatal screening excluded an SDM focus, it covered ethical aspects of the decision and information on the importance of adapting prenatal care to each pregnant woman, a core value in the nursing profession [57,58]. Consequently, it is possible that there was not enough difference between the study arms in terms of generic decision support for prenatal care. Indeed, previous research has shown that of all health professions, genetic counselors have the highest SDM scores during prenatal consultations, even in the absence of SDM training [59,60]. In addition, we observed a ceiling effect with the primary outcome at baseline, which may have limited our ability to observe the effect of the intervention. Similar ceiling effects were seen in a study assessing change in behavioral intention after CPD activities [48] and in another study assessing the impact of a patient–professional coproduced digital educational intervention [61]. Our initial high scores may also be because of the training that Canadian nurses had already received. Nursing programs emphasize fundamental knowledge of relational approaches that influence health outcomes for individuals, families, and communities [57]. In fact, the essence of nursing is based on a deep understanding of the biopsychosocial aspects of patients and on advocating for them [62]. Finally, participants may have been subjected to social pressure to state that they intended to use a DA [49,63-65]. Thus, we do not have insight into the full set of factors that may have contributed to their intention (apart from the CPD-Reaction constructs), because the theories explain only part of the variance in intention [46].

Second, we found a statistically significant difference in scores for knowledge about SDM between the intervention and control groups but not for knowledge about the topics covered in both arms, such as prenatal screening and Down syndrome. Thus, the intervention arm (the SDM training program) did distinguish itself from transmitting knowledge about SDM. In addition, the intervention group found their course more acceptable and had a more positive reaction to its pedagogical approach than did the control group. This can be explained in part by the speakers, as participants in the control group expressed difficulties in understanding the speaker in their course. The course format may also have made a difference. The SDM course included a DA and a simulation video, which, especially in the context of web-based pedagogy and regardless of its content, may have been a more acceptable and effective format than the control course [37].

Third, we did not measure whether this high level of intention translated into behavior. Although intention is a strong predictor of behavior [46], the implementation of SDM in practice might depend on other external variables, such as organizational limitations [26]. Indeed, in their written comments, nurses mentioned that organizational barriers prevented them from using DAs in their clinical setting, regardless of their intention, and that they had little time to share decisions with their patients given the speed of lead up to prenatal screening procedures. Another organizational barrier that could defeat nurses’ best intentions may be related to the role they are assigned in interprofessional teams or their ability to collaborate with other professionals to ensure that SDM occurs [28]. The organizational environment and care pathways need to be adapted so that pregnant women and nurses can engage fully in SDM, which is already a good fit with nurses’ role in the health care system.

### Limitations

This study has several limitations. First, in contrast with the planned study design, randomization allocation was not implemented. The private firm, unbeknownst to us, instead focused on ensuring a balanced number of participants in both arms by allocating them alternately to one group or the other. Nevertheless, the study was controlled and allowed us to compare the intervention and control groups. Second, nurses who agreed to participate in this trial may not be representative of all nurses involved in prenatal care in Quebec because of our web-based recruitment methods. For example, in this study, there was only 1 nurse practitioner who had a different scope of practice than other nurses. In addition, our findings may not be generalizable to nurses’ engagement in SDM in other areas of their practice, such as disease management. Prenatal care does not usually involve disease management, as pregnancy itself is not an illness. Third, these results cannot be generalized to other health professionals involved in prenatal care in Quebec, as studies show that each approaches the topic of SDM in a different way [16,53]. Training nurses using an interprofessional approach to SDM might encourage interprofessional interaction and information exchange about SDM [28]. Exploration of an interdisciplinary DA model will be examined in future research.

### Conclusions

This study focuses on the potential of web-based nursing education to support pregnant women’s decision-making needs. We assessed nurses’ intention to use a DA to support prenatal screening decisions among pregnant women and their overall impressions regarding the training. This study showed that nurses’ intention to use a DA in prenatal care is already high, with or without training, but that their knowledge about SDM could be improved with training. Our results will inform future strategies for implementing SDM behaviors in this population. The study also suggested avenues for future evaluations of SDM training programs. The next steps will be to (1) update the program by incorporating the written comments received from the participants and (2) evaluate the impact of the course with all health professionals involved in prenatal care.

## Appendix

Multimedia Appendix 1 CONSORT-eHEALTH checklist (V 1.6.1).

Multimedia Appendix 2 Report of both programs according to the Template for Intervention Description and Replication items.

Multimedia Appendix 3 Screenshots of the training content.

Multimedia Appendix 4 Data collection questionnaire after the training.

## Abbreviations

CONSORT-EHEALTH: Consolidated Standards of Reporting Trials of Electronic and Mobile Health Applications and Online Telehealth
CPD: continuing professional development
DA: decision aid
PEGASUS: Personalized Genomics for Prenatal Abnormalities Screening Using Maternal Blood
SDM: shared decision-making
TIDieR: Template for Intervention Description and Replication items
