# Supplementary material for: Web-Based Training for Nurses on Using a Decision Aid to Support Shared Decision-making About Prenatal Screening: Parallel Controlled Trial
Source: JMIR Nurs. 2022 Jan 25;5(1):e31380. doi: 10.2196/31380 (PMC8826152; doi:10.2196/31380)
Supplement: Multimedia Appendix 2 [file nursing_v5i1e31380_app2.pdf]

Supplementary file - Reporting of both programs according to the TIDieR items

TIDieR item number	TIDieR item name	Intervention Group	Control Group
**1**	**Brief name**	Web-Based Training Program on SDM and Down Syndrome Prenatal Screening	Web-Based Training Program and Down Syndrome Prenatal Screening
**2**	**Why**	This training on shared decision making aims to improve understanding of and knowledge about trisomy 21 screening. It primarily targets health professionals involved in prenatal follow-up of pregnant women and who support them in the decision about whether to take a prenatal screening test for trisomy 21. It is also relevant for anyone interested in shared decision-making, the use of decision support tools or prenatal screening.	This training aims to improve the understanding of issues surrounding the screening for trisomy 21. It is mainly aimed at health professionals who are involved in prenatal follow-up of pregnant women and who support them in the decision about whether to take a prenatal screening test for trisomy 21. It is also relevant for anyone interested in the ethics of prenatal screening.
**3**	**What (materials)**	Four modules containing videos, narrative capsules, additional resources (e.g. scientific articles and relevant links), audio of the videos, and a simulation module.<br>To access the training, the learner must have or be issued a Laval University ID.	Four modules containing videos, narrative capsules and additional resources (e.g. scientific articles and relevant links).<br><br>To access the training, the learner must have or be issued a Laval University ID.
**4**	**What (procedures)**	Once participants have access to the training, they complete the modules including the simulation module and fill out the questionnaires.	Once participants have access to the training, they complete the modules and fill out the questionnaires.
**5**	**Who provided**	Self-training	Self-training
**6**	**How**	Online	Online
**7**	**Where**	The training can be done anywhere - At the participant's preferred location.	The training can be done anywhere - At the participant's preferred location.
**8**	**When and how much**	Once, at any time according to the availability of the participant.	Once, at any time according to the availability of the participant.
**9**	**Tailoring**	Not applicable	Not applicable
**10**	**Modifications**	No changes were made to the training during the intervention.	No changes were made to the training during the intervention.
**11**	**Fidelity (Planned) – If assessed**	Not assessed	Not assessed
**12**	**Fidelity (Actual) – If assessed**	Not assessed	Not assessed
