# Supplementary material for: Web-Based Training for Nurses on Using a Decision Aid to Support Shared Decision-making About Prenatal Screening: Parallel Controlled Trial
Source: JMIR Nurs. 2022 Jan 25;5(1):e31380. doi: 10.2196/31380 (PMC8826152; doi:10.2196/31380)
Supplement: Multimedia Appendix 3 [file nursing_v5i1e31380_app3.pdf]

Formation : Prise de décision partagée pour le test de dépistage prénatal de la trisomie 21

Ce site n’est pas encore publié. Il est présentement accessible uniquement à l’enseignant et aux assistants, s’il y a lieu.

Introduction

Mot de bienvenue

Nous vous souhaitons la bienvenue à cette formation en ligne sur la « Prise de décision partagée pour le test de dépistage prénatal de la trisomie 21 ».

Cette formation sur la prise de décision partagée vise à améliorer la compréhension et les connaissances dans le contexte du dépistage de la trisomie 21. Elle cible principalement les professionnels de la santé qui sont impliqués dans le suivi prénatal des femmes enceintes et qui les soutiennent dans la décision de recourir ou non au dépistage prénatal de la trisomie 21. Elle est également pertinente pour toute personne qui s’intéresse à la prise de décision partagée, à l’utilisation d’outils d’aide à la décision ou encore au dépistage prénatal.

Bonne formation !

Dre France Légaré, B. Arch, MD, PhD, CCMF, FCMF

Présentation de la formation

Liens du projet de recherche

- Projet de recherche PEGASUS
- Équipe de la Chaire de recherche du Canada sur la décision partagée et l’application des

connaissances

Images

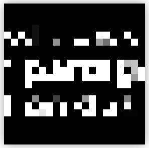

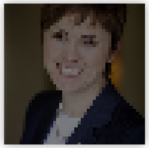

Attestation

Il est possible de commander une attestation de particiation pour cette formation.

Vous trouverez les détails à la fin de la formation.

© Université Laval, 2019. Tous droits réservés.

Formation : Prise de décision partagée pour le test de dépistage prénatal de la trisomie 21

Ce site n'est pas encore publié. Il est présentement accessible uniquement à l'enseignant et aux assistants, s'il y a lieu.

Description de la formation

> Descriptif
> Objectifs d'apprentissage
> Structure de la formation
> Formules pédagogiques
> Évaluation
> Période d'accès et attestation

Descriptif

Faire ou ne pas faire un test de dépistage prénatal pour évaluer le risque du foetus d'être atteint de certaines maladies génétiques est une décision difficile à laquelle sont confrontés toutes les femmes enceintes et leurs partenaires. Il est essentiel que les femmes enceintes et leurs partenaires soient adéquatement informés sur les avantages, les inconvénients et les risques de ces tests de dépistage et qu'ils soient soutenus dans la prise de telles décisions. Pour ce faire, plusieurs stratégies peuvent être mises en place, telle que l'utilisation d'outils d'aide à la décision qui permettent aux femmes enceintes et à leurs partenaires de s'engager dans un processus de prise de décision partagée. Après avoir complété cette formation en ligne, vous serez en mesure d'utiliser un outil d'aide à la décision et de vous engager dans un processus de prise de décision partagée avec les femmes enceintes et leurs partenaires dans le contexte du dépistage prénatal de la trisomie 21.

Objectifs d'apprentissage

Objectif général : Utiliser l’outil d’aide à la décision en contexte de suivi prénatal

Objectifs spécifiques :

1. Définir le concept de décision partagée
2. Nommer les objectifs du dépistage prénatal de la trisomie 21
3. Identifier les points de décision dans le contexte du dépistage prénatal de la trisomie 21
4. Identifier les principales caractéristiques qui distinguent un outil d’aide à la décision d’un dépliant d’information
5. Appliquer des stratégies efficaces afin de communiquer les options associées au dépistage prénatal de la trisomie 21 ainsi que les avantages et inconvénients qui y sont reliés
6. Utiliser des stratégies efficaces afin d’identifier les valeurs et les préférences des patientes dans le contexte du dépistage prénatal de la trisomie 21

Structure de la formation

Pour faciliter votre apprentissage, la formation en ligne a été divisée en quatre modules principaux :

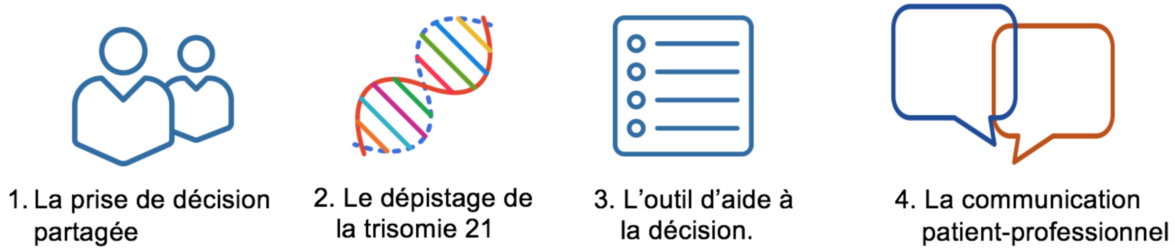

Dans chaque module, vous trouverez les objectifs d'apprentissage visés ainsi que le travail à réaliser (ex. lectures à faire, vidéo à visionner, évaluation à réaliser, etc.). Nous vous conseillons fortement de suivre l’ordre de présentation des différents modules établis dans la section « Contenus et activités » puisque la séquence d’apprentissage proposée a été établie pour favoriser un apprentissage progressif. Également, dans chaque module, vous remarquerez la présence d'une section nommée «En savoir plus ...». Bien que la consultation des ressources complémentaires soit facultative, ces ressources sont mises à votre disposition pour vous permettre d'approfondir, au besoin, divers aspects liés aux thèmes abordés dans cette formation.

Lorsque vous aurez complété l’ensemble des activités qui composent la formation, vous serez appelé à effectuer une tâche intégratrice sous forme d’une « Simulation ». Cette simulation vidéo a pour but de vous immerger dans une situation très

près de la réalité vécue par les professionnels de la santé qui offrent des soins et des services aux femmes enceintes. Par le biais de cette activité, vous serez amené à mettre en application les apprentissages que vous aurez réalisés lors de la formation.

Finalement, vous êtes chaleureusement invité à prendre quelques minutes pour compléter le questionnaire d'appréciation de la formation qui est disponible dans la section « Appréciation de la formation ». Vos commentaires sont très précieux pour l'amélioration de la formation.

Formules pédagogiques

Différentes méthodes pédagogiques ainsi que des médias variés seront utilisés au cours de cette formation: vidéos, entrevues filmées, capsules narrées, lectures, liens vers des articles scientifiques et des sites web complémentaires. Également, de courts questionnaires vous permettront de vérifier vos apprentissages et une simulation vidéo vous amènera à mettre en application les apprentissages que vous aurez réalisés lors de la formation.

Cette formation a été conçue pour s'adapter à votre rythme d'apprentissage, ainsi vous n'êtes pas obligé de la réaliser en continu. Si vous quittez le site de formation, vous pourrez y revenir plus tard et reprendre la formation là où vous étiez rendu. À tout moment, il est également possible de revoir les contenus associés aux différentes sections de la formation au besoin.

Évaluation

Une évaluation formative est prévue à la fin de chaque module afin de valider les notions abordées. Celle-ci est sous forme de questions à choix multiples.

Période d'accès et attestation

La durée de cette formation en ligne est estimée à 3 heures et compte pour

- 3 crédits de catégorie 1 de formation continue pour les médecins;
- 0,3 unité d'éducation continue (UEC) pour tous les autres professionnels de la santé.

À la suite de votre formation, vous avez l'opportunité de commander votre attestation de participation. Pour en savoir plus sur la commande de votre attestation, je vous invite à consulter la rubrique « Attestation » affichée après la simulation vidéo. De plus, vous conserverez vos accès à cette formation 90 jours suivant votre inscription.

Formation : Prise de décision partagée pour le test de dépistage prénatal de la trisomie 21

Contenu et activités

Titre	Date
Modules de formation	
1. La décision partagée	
2. Le dépistage prénatal de la trisomie 21	
3. L'outil d'aide à la décision	
4. La communication entre le professionnel et le patient	
Mise en application	
Simulation	
Appréciation de la formation	
Évaluation du programme	
Forum de discussion	
Forum de discussion	
Section pour les collaborateurs	

© Université Laval, 2019. Tous droits réservés.

Formation : Prise de décision partagée pour le test de dépistage prénatal de la trisomie 21

1. La décision partagée

Activités | Évaluation | En savoir plus ... | Votre expert

Télécharger les fichiers

Objectifs d'apprentissage

Au terme de ce module, vous serez en mesure de :

- Définir le concept de décision partagée
- Reconnaître la pertinence de la décision partagée
- Identifier les éléments favorisant ou entravant l'implantation de la décision partagée dans les milieux cliniques
- Énumérer les avantages pour les professionnels de la santé et les patients

À faire

Dans ce module, nous vous invitons à :

1. Visionner les vidéos ci-dessous et lire les documents associés.
2. Compléter le questionnaire qui apparaît sous l'onglet *Évaluation*.

1. Le concept de décision partagée

Modèles de prise de décision
547,35 Ko, déposé le 18 déc. 2018

2. Pourquoi et quand est-ce pertinent?

Exemples de surutilisation
562,19 Ko, déposé le 18 déc. 2018

3. Éléments favorisant/entravant l'implantation dans les milieux cliniques?

4. Avantages pour les patients et les professionnels de la santé

5. La prise de décision partagée au cœur du suivi prénatal

Communiquer le risque en matière de dépistage prénatal, pas un jeu d'enfant!
208,25 Ko, déposé le 7 déc. 2018

Impacts de la prise de décision partagée
378,06 Ko, déposé le 18 déc. 2018

Versions audio des capsules vidéo

1. Le concept de décision partagée
7,12 Mo, déposé le 19 déc. 2018

2. Pourquoi et quand est-ce pertinent?
12,25 Mo, déposé le 19 déc. 2018

3. Éléments favorisant/entravant l'implantation dans les milieux cliniques?
12,71 Mo, déposé le 19 déc. 2018

4. Avantages pour les patients et les professionnels de la santé
9,68 Mo, déposé le 19 déc. 2018

5. La prise de décision partagée au cœur du suivi prénatal
5,91 Mo, déposé le 19 déc. 2018

© Université Laval, 2019. Tous droits réservés.

Formation : Prise de décision partagée pour le test de dépistage prénatal de la trisomie 21

1. La décision partagée

Activités | **Évaluation** | En savoir plus ... | Votre expert

Directives

Pour démontrer que vous avez bien atteint les objectifs visés par ce module, vous devez répondre à un questionnaire formatif en ligne. Pour y accéder, cliquez sur le lien ci-dessous.

Préparation à l'évaluation

- Ce questionnaire formatif porte sur l'ensemble du contenu couvert par le module 1 : *La décision partagée* (document et vidéos), excluant le matériel sous l'onglet *En savoir plus...* .
- Avant de débuter le questionnaire, relisez les objectifs mentionnés sous l'onglet *Contenus* et assurez-vous que vous êtes en mesure de réaliser ce qui est attendu au terme de ce module.

Questionnaire - module 1 (en entier) (À faire)
Disponible en tout temps
Tentatives : 0 / 3

© Université Laval, 2019. Tous droits réservés.

Formation : Prise de décision partagée pour le test de dépistage prénatal de la trisomie 21

1. La décision partagée

Activités Évaluation En savoir plus ... Votre expert

Télécharger les fichiers

Envie d'en savoir plus?

Voici des suggestions de ressources complémentaires en lien avec le thème de ce module. Bien que la consultation des ressources complémentaires soit facultative, ces ressources pourront vous permettre d'approfondir divers aspects liés au thème abordé au cours de ce module.

- Site web de la Chaire de recherche du Canada Niveau 1 sur la prise de décision partagée et l'application des connaissances

Chaire de recherche du Canada en décision partagée et application de connaissances

- Article décrivant les éléments clés et les barrières à l'adoption de la prise de décision partagée

Shared Decision Making: Examining Key Elements And Barriers To Adoption Into Routine Clinical Practice
182,1 Ko, déposé le 13 nov. 2018

- Article qui présente les mythes associés à la décision partagée

Twelve myths about shared decision making
372,52 Ko, déposé le 13 nov. 2018

- Sommaire de la revue Cochrane sur les interventions pour améliorer l'adoption de la prise de décision partagée

Interventions for increasing the use of shared decision making by healthcare professionals
119,71 Ko, déposé le 13 nov. 2018

© Université Laval, 2019. Tous droits réservés.

< Cours

Formation : Prise de décision partagée pour le test de dépistage prénatal de la trisomie 21

1. La décision partagée

Activités Évaluation En savoir plus ... Votre expert

Télécharger les fichiers

Dre France Légaré

Experte sur la décision paratgée

Professeure

Mme France Légaré

Clinicienne - Chercheuse
Centre de recherche sur les soins et les services de première ligne de l'Université La val

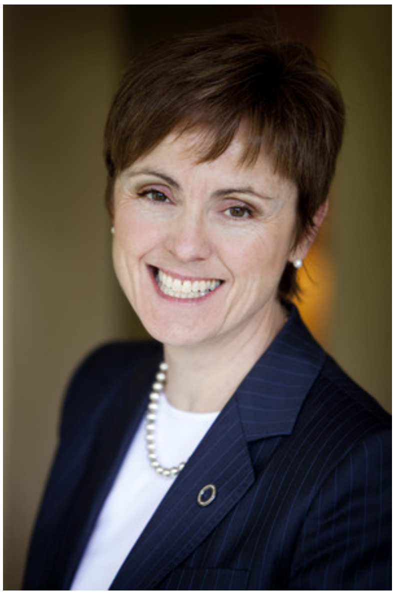

Description

Initialement formée comme architecte et par la suite formée comme médecin de famille à Québec avec une maîtrise en santé communautaire, France Légaré est professeure titulaire au département de médecine familiale et de médecine d'urgence de l'Université Laval. En 2005, elle obtient son doctorat en santé des populations à l'Université d'Ottawa sous la supervision de la Dre Annette O'Connor. Cette même année, elle obtient une bourse de chercheur clinicien du Fonds de la recherche en santé du Québec (FRSQ) pour son programme de recherche : « Professionnels de la santé en soins primaires : de courtiers des connaissances à courtier des décisions ».

Entre juin 2006 et mai 2016, elle a détenu le titre de titulaire de la Chaire de recherche du Canada sur la décision partagée et l'application des connaissances, niveau junior. Depuis le 1er juin 2016, elle détient le titre de titulaire Tier 1 de la Chaire de recherche du Canada sur la prise de décision partagée et l'application des connaissances. Également, France a dirigé le site Cochrane du CRCHUQ et de l'Université Laval au sein du réseau canadien Cochrane de 1999 à 2013, et de 2013-15 comme première directrice scientifique. Elle agit maintenant comme co-directrice scientifique.

- Directrice, composante « Recherche sur les systèmes de santé et services sociaux, sur l'application des connaissances et sur la mise-en-œuvre », Unité de SOUTIEN-SRAP du Québec
- Clinicienne enseignante, UMF Saint-François d'Assise
- Chercheuse associée, Programme d'Épidémiologie Clinique, Institut de recherche de l'Hôpital d'Ottawa (IRHO)

- Chercheuse régulière au Centre de recherche sur les soins et les services de premières lignes de l’université Laval (CERSSPL-UL)

© Université Laval, 2019. Tous droits réservés.

Formation : Prise de décision partagée pour le test de dépistage prénatal de la trisomie 21

2. Le dépistage prénatal de la trisomie 21

Objectifs d'apprentissage

Au terme de ce module, vous serez en mesure de :

- Nommer les objectifs du dépistage prénatal de la trisomie 21.
- Identifier les points de décision dans le contexte québécois du dépistage prénatal de la trisomie 21.
- Identifier les enjeux éthiques et sociaux du dépistage prénatal.

À faire

Dans ce module, nous vous invitons à :

1. Visionner les 3 vidéos avec nos expert, Dr Francois Rousseau et Dr Jean-Claude Forest, sur le dépistage prénatal et consulté les documents associés.

2. Visionner les 2 vidéos de notre expert, Dre Vardit Ravitsky, sur les considérations éthiques.

3. Compléter le questionnaire qui apparaît sous l'onglet *Évaluation*.

1. Caractéristiques des trisomies 13, 18 et 21 et le dépistage prénatal

2. Programme québécois de dépistage prénatal de la trisomie 21

3. Dépistage prénatal et l'outil d'aide à la décision

Étapes du Programme Québécois de dépistage prénatal de la trisomie 21
362,33 Ko, déposé le 18 déc. 2018

4. Les enjeux éthiques et sociaux du dépistage prénatal

Arbre décisionnel du Programme Québécois de dépistage prénatal de la trisomie 21
333,26 Ko, déposé le 18 déc. 2018

Arbre décisionnel futur incluant le test de dépistage prénatal non-invasif en 2ième intention
341,88 Ko, déposé le 18 déc. 2018

5. Avantages et désavantages de la prise de décision partagée

Versions audio des capsules vidéo

1. Caractéristiques des trisomies 13, 18 et 21 et le dépistage prénatal
7,81 Mo, déposé le 19 déc. 2018

2. Programme québécois de dépistage prénatal de la trisomie 21
14,31 Mo, déposé le 19 déc. 2018

3. Dépistage prénatal et l'outil d'aide à la décision
12,09 Mo, déposé le 19 déc. 2018

4. Les enjeux éthiques et sociaux du dépistage prénatal
16,57 Mo, déposé le 19 déc. 2018

5. Avantages et désavantages de la prise de décision partagée
10,7 Mo, déposé le 19 déc. 2018

© Université Laval, 2019. Tous droits réservés.

Formation : Prise de décision partagée pour le test de dépistage prénatal de la trisomie 21

2. Le dépistage prénatal de la trisomie 21

Activités | Évaluation | En savoir plus ... | Vos experts

Directives

Pour démontrer que vous avez bien atteint les objectifs visés par ce module, vous devez répondre à un questionnaire formatif en ligne. Pour y accéder, cliquez sur le lien ci-dessous.

Préparation à l'évaluation

- Ce questionnaire formatif porte sur l'ensemble du contenu couvert par le module 2 : *Le dépistage prénatal de la trisomie 21* (documents et vidéos), excluant le matériel sous l'onglet *En savoir plus...* .
- Avant de débuter le questionnaire, relisez les objectifs mentionnés sous l'onglet *Contenus* et assurez-vous que vous êtes en mesure de réaliser ce qui est attendu au terme de ce module.

Questionnaire du module 2 (À faire)
Disponible en tout temps
Tentatives : 0 / 1

© Université Laval, 2019. Tous droits réservés.

Formation : Prise de décision partagée pour le test de dépistage prénatal de la trisomie 21

2. Le dépistage prénatal de la trisomie 21

Envie d'en savoir plus?

Voici des suggestions de ressources complémentaires en lien avec le thème de ce module. Bien que la consultation des ressources complémentaires soit facultative, ces ressources pourront vous permettre d'approfondir divers aspects liés au thème abordé au cours de ce module.

- Programme québécois de dépistage prénatal de la trisomie 21

Programme québécois de dépistage prénatal de la trisomie 21

Formation du Programme québécois de dépistage prénatal de la trisomie 21

- Revue systématique identifiant les besoins décisionnels de femmes enceintes, de leur partenaire et des professionnels de la santé en matière de dépistage prénatal

Decisional needs assessment regarding Down syndrome prenatal testing: a systematic review of the perceptions of women, their partners and health professionals
149,01 Ko, déposé le 13 nov. 2018

- Sommaire de la revue Cochrane estimant la précision des tests de dépistage prénatal de premier et second trimestre

First and second trimester serum tests with and without first trimester ultrasound tests for Down's syndrome screening
114,66 Ko, déposé le 13 nov. 2018

- Article soulignant l'importance que les femmes enceintes prennent des décisions éclairées en matière de dépistage prénatal

Supporting Women's Autonomy in Prenatal Testing
365,15 Ko, déposé le 13 nov. 2018

- Rapport d'une consultation sur les enjeux éthiques du dépistage prénatal de la trisomie 21 au Québec

Consultation sur les enjeux éthiques du dépistage prénatal de la trisomie 21, ou syndrome de Down, au Québec
5,26 Mo, déposé le 13 nov. 2018

< Cours

Formation : Prise de décision partagée pour le test de dépistage prénatal de la trisomie 21

2. Le dépistage prénatal de la trisomie 21

Activités Évaluation En savoir plus ... Vos experts

Télécharger les fichiers

Dr François Rousseau

Expert sur le dépistage prénatal

Professeur

M. Francois Rousseau

Médecin Biochimiste
Centre de recherche du CHU de Québec-Université Laval, Hôpital Saint-François d'A ssise

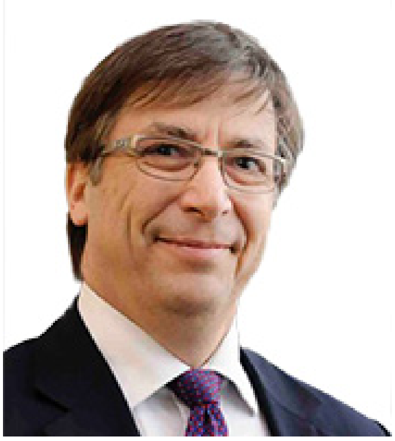

Vidéo de présentation

Dr Rousseau
5,78 Mo, déposé le 19 déc. 2018

Le Dr François Rousseau est un médecin biochimiste surspécialisé en génétique moléculaire humaine. Il a obtenu un baccalauréat en médecine (1983), un doctorat en médecine (1984) et une maîtrise en ontogénétique moléculaire (1987), tous de l'Université Laval. Il a complété une formation postdoctorale de trois ans en génétique moléculaire humaine à l'Université Louis-Pasteur de Strasbourg (1989-1991), où il a contribué à la découverte du gène du syndrome de l'X fragile, cause la plus fréquente de retard mental héréditaire.

Il est membre expert de plusieurs comités nationaux et internationaux sur les tests de diagnostic, notamment pour l'Institut national d'excellence en soins et services sociaux (INESSS) où il préside le comité scientifique des analyses de biologie médicale. Il est auteur de plus de 130 publications scientifiques ayant donné lieu à plus de 10 000 citations, et est éditeur associé du plus important manuel de médecine de laboratoire et de diagnostic moléculaire. Il a présidé, pendant le terme maximal de 6 ans, le comité de diagnostic moléculaire de la Fédération internationale de médecine de laboratoire (IFCC), regroupant plus de 80 pays. Il est Fellow de l'Académie Canadienne des sciences de la santé depuis 2014. Depuis 2005, il dirige le département de médecine de laboratoire du CHU de Québec-Université Laval qui compte plus de 110 médecins, 600 technologistes médicaux et produit 11 % de tous les tests de laboratoire de la province de Québec.

Ses travaux de recherche ont porté sur la prévalence des mutations du gène du syndrome X-fragile dans la population générale, puis sur les données probantes nécessaires au transfert vers la médecine clinique des découvertes issues de la génétique et la génomique humaines. Il est co-fondateur du Laboratoire de simulation économique de dépistage qui permet d'évaluer le coût/efficacité et le coût/utilité des innovations, afin d'informer les décideurs de notre système de santé.

Il a été chercheur principal désigné du consortium de recherche (30 chercheurs et 4 pays) APOGEE-Net/CanGeneTest financé par les IRSC sur la recherche sur les services de santé en génétique (2005-2013), axé sur la validation et le transfert d'innovations génétiques et génomiques cliniquement utiles et rentables pour le système de santé.

Il est également le leader du projet PÉGASE I et PÉGASE II financé par Génome Canada (30 chercheurs, 5 pays, 8 universités canadiennes et 4 universités à l'étranger) sur l'efficacité comparative réelle des technologies génomiques pour le dépistage prénatal non-invasif. Il est également visiteur en accréditation de laboratoire clinique pour Agrément Canada, depuis 2009. Ses recherches portent sur la traduction des découvertes issues du projet du génome humain dans le système de soins de santé, ainsi que dans la médecine de laboratoire fondée sur des données probantes, l'évaluation des technologies génomiques, et leur valeur pour le système de santé.

Dr Jean-Claude Forest

Expert sur le dépistage prénatal québécois

Professeur

M. Jean-Claude Forest

Clinicien - Chercheur
Centre de recherche du CHU de Québec - Université Laval, Hôpital Saint-François d'Assise

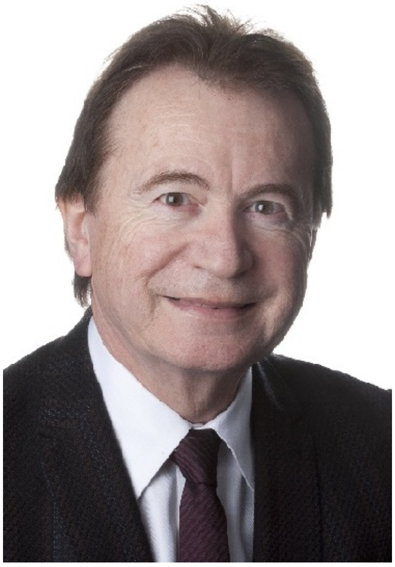

Vidéo de présentation

Dr Forest
3,43 Mo, déposé le 19 déc. 2018

Le Dr Jean-Claude Forest est chercheur-clinicien, membre de l'axe « Reproduction, santé de la mère et de l'enfant » du Centre de recherche du CHU de Québec-Université Laval. Il est professeur titulaire de biochimie médicale à la Faculté de médecine de l'Université Laval et membre du Département de médecine de laboratoire du CHU de Québec. Il a été directeur du Centre de recherche du Centre hospitalier universitaire de Québec (CHUQ) de 2004 à 2011, et directeur général adjoint, affaires médicales et universitaires du CHU de Québec depuis 2010.

Ses principaux intérêts de recherche visent le développement de marqueurs biologiques en vue du dépistage et du diagnostic d'anomalies fœtales et de maladies associées à la grossesse, telles la prééclampsie et le diabète gestationnel. Il est l'auteur ou le coauteur de plus de 600 articles, chapitres de livres, communications scientifiques et conférences sur invitation. Ses travaux ont contribué, entre autres, à la mise sur pied de programmes de dépistage prénatal de la trisomie 21.

D'ailleurs, il préside, le comité de coordination du programme québécois auquel il a activement participé au déploiement. De 1994 à 2006, il a été successivement membre, vice-président et président de la Division scientifique de la Fédération internationale de chimie clinique et de médecine de laboratoire (IFCC). Il a présidé, de 2006 à 2009, au sein du Bureau International des Poids et Mesures, le comité sur la traçabilité métrologique en médecine de laboratoire et, de 2005 à 2010, il a été délégué au comité d'experts de l'Organisation Mondiale de la Santé (OMS) sur la standardisation biologique. Le Dr Forest est membre de nombreuses associations médicales et savantes.

Il a reçu de nombreux prix et distinctions, tant sur la scène nationale qu'internationale. À titre d'exemple, en 2017, il a été le récipiendaire de l'une des plus hautes distinctions de l'Association médicale du Canada, la Médaille de service et du prix « *IFCC- Abbott Visiting Lecturer Award* » de l'IFCC.

Dre Vardit Ravitsky

Experte en Bioéthique

Professeure

Mme Vardit Ravitsky

Professeure agrégeé aux programmes de bioéthiques
Département de médecine sociale et préventive de l'École de santé publique de l'U
de Montréal

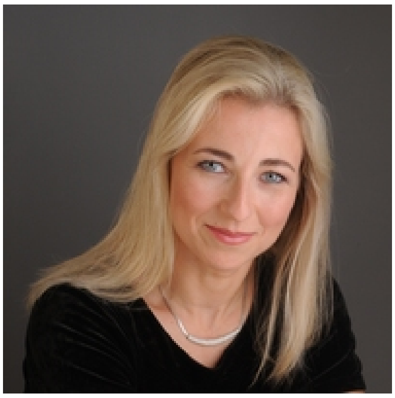

Vidéo de présentation

Dre Ravitsky
6,35 Mo, déposé le 19 déc. 2018

Vardit Ravitsky est professeure agrégeé aux programmes de bioéthiques, département de médecine sociale et préventive de l'École de santé publique de l'Université de Montréal (ÉSPUM). Elle est également membre de l'Institut de recherche en santé publique de l'Université de Montréal (IRSPUM). Elle a fait ses études en France, Etats-Unis et Israël. Après une formation post doctorale à l'NIH, elle a occupé un poste académique à l'Université de la Pennsylvanie (USA), un poste de Consultante au Génome Canada et un poste de Conseillère à l'Instituts de recherche en sante du Canada (IRSC). Ses enseignements portent sur l'éthique clinique et l'éthique de la procréation. Ses intérêts de recherche concernent les enjeux bioéthiques de la procréation médicalement assistée et de la génétique. Elle s'intéresse particulièrement aux influences culturelles sur le développement des politiques de santé autours des dilemmes bioéthiques.

© Université Laval, 2019. Tous droits réservés.

< Cours

Formation : Prise de décision partagée pour le test de dépistage prénatal de la trisomie 21

3. L'outil d'aide à la décision

Activités Évaluation En savoir plus ...

Télécharger les fichiers

Objectifs d'apprentissage

Au terme de ce module, vous serez en mesure de :

- Identifier les points de décision dans le contexte du dépistage prénatal de la trisomie 21.
- Identifier les principales caractéristiques qui distinguent un outil d'aide à la décision d'un dépliant d'information.

1. L'utilisation d'un outil d'aide

2. Capsule interactive

Explorer l'outil d'aide à la décision utilisé dans le contexte du dépistage prénatal de la trisomie 21 .

À faire

Dans ce module, nous vous invitons à :

1. Visionner la vidéo de Dr D'Amours
2. Visionner la capsule interactive expliquant les différentes caractéristiques de l'outil d'aide à la décision.
3. Prendre connaissance des outils d'aide à la décision.
    - Dans une clinique médicale publique
    - Dans une clinique médicale privée
    - Dans une clinique de procréation assistée

4. Compléter le questionnaire qui apparaît sous l'onglet *Évaluation*.

3. Outils d'aide à la décision

Pour clinique médicale publique (avant avril 2019).

Outil d'aide à la décision n'offrant pas le test de dépistage non-invasif.
5,2 Mo, déposé le 13 nov. 2018

Pour clinique médicale publique (à partir d'avril 2019) et clinique médicale privée.

Outil d'aide à la décision incluant le test de dépistage prénatal non-invasif en deuxième intention.
1,4 Mo, déposé le 13 nov. 2018

Pour clinique de procréation.

Outil d'aide à la décision incluant le test de dépistage prénatal non-invasif en première intention.
1,16 Mo, déposé le 13 nov. 2018

Version audio de la capsule vidéo

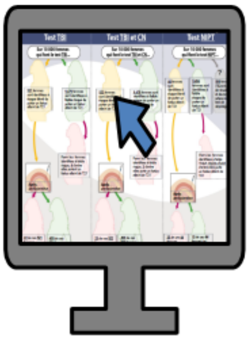

Outil d'aide

Aucun contenu à afficher pour l’instant.

© Université Laval, 2019. Tous droits réservés.

Formation : Prise de décision partagée pour le test de dépistage prénatal de la trisomie 21

3. L'outil d'aide à la décision

Activités | Évaluation | En savoir plus ...

Directives

Pour démontrer que vous avez bien atteint les objectifs visés par ce module, vous devez répondre à un questionnaire formatif en ligne. Pour y accéder, cliquez sur le lien ci-dessous.

Préparation à l'évaluation

- Ce questionnaire formatif porte sur l'ensemble du contenu couvert par le module 3 : *L'outil d'aide à la décision* (documents et vidéo), excluant le matériel sous l'onglet *En savoir plus...* .
- Avant de débuter le questionnaire, relisez les objectifs mentionnés sous l'onglet *Contenus* et assurez-vous que vous êtes en mesure de réaliser ce qui est attendu au terme de ce module.

Questionnaire du module 3 (À faire)
Disponible en tout temps
Tentatives : 0 / 1

© Université Laval, 2019. Tous droits réservés.

Formation : Prise de décision partagée pour le test de dépistage prénatal de la trisomie 21

3. L'outil d'aide à la décision

Envie d'en savoir plus?

Voici des suggestions de ressources complémentaires en lien avec le thème de ce module. Bien que la consultation des ressources complémentaires soit facultative, ces ressources pourront vous permettre d'approfondir divers aspects liés au thème abordé au cours de ce module.

- Sommaire de la revue Cochrane évaluant l'effet des outils d'aide à la décision sur les individus faisant face à des décisions de traitement ou de dépistage

Decision aids for people facing health treatment or screening decisions
3,5 Mo, déposé le 13 nov. 2018

- Grille des critères internationaux pour les outils d'aide à la décision s'adressant aux patients - "International Patient Decision Aid Standards" (IPDAS)

International Patient Decision Aid Standards (IPDAS)
86,18 Ko, déposé le 13 nov. 2018

- Facteurs qui influencent l'intention des professionnels de la santé d'utiliser un outil d'aide à la décision dans le contexte du dépistage prénatal de la trisomie 21

What factors influence health professionals to use decision aids for Down syndrome prenatal screening.pdf
446,16 Ko, déposé le 13 nov. 2018

- Facteurs qui influencent l'intention des femmes enceintes d'utiliser un outil d'aide à la décision dans le contexte du dépistage prénatal de la trisomie 21

Use of a patient decision aid for prenatal screening for Down syndrome_what do pregnant women say.pdf
448,18 Ko, déposé le 13 nov. 2018

Matériel supplémentaire pour des objectifs de recherche

- Revue systématique des données probantes qui composent les outils d'aide à la décision

What information is used in treatment decision aids?
527,79 Ko, déposé le 13 nov. 2018

- Cartographie des outils d'aide à la décision qui supportent les décisions sur le dépistage de la trisomie 21

Decision aids that support decisions about prenatal testing for Down syndrome
643,2 Ko, déposé le 13 nov. 2018

- Critères internationaux pour les outils d'aide à la décision s'adressant aux patients - "International Patient Decision Aid Standards" (IPDAS)

http://ipdas.ohri.ca/

- Outil d'aide à la décision pour les patients - Répertoire des outils d'aide à la décision

https://decisionaid.ohri.ca/francais/repertoire.html

- Outil d'aide à la décision pour les patients - Méthodes de développement

https://decisionaid.ohri.ca/francais/methode-dev.html

© Université Laval, 2019. Tous droits réservés.

< Cours

Formation : Prise de décision partagée pour le test de dépistage prénatal de la trisomie 21

4. La communication entre le professionnel et le patient

Activités | Évaluation | En savoir plus ... | Votre expert

Télécharger les fichiers

Objectifs d'apprentissage

Au terme de ce module, vous serez en mesure de :

- Appliquer des stratégies efficaces afin de communiquer les options associées au dépistage prénatal de la trisomie 21 ainsi que les avantages et inconvénients qui y sont reliés.
- Utiliser des stratégies efficaces afin d'identifier les valeurs et les préférences des patientes dans le contexte du dépistage prénatal de la trisomie 21.

1. La communication entre le professionnel de la santé et le patient

À faire

Dans ce module, nous vous invitons à :

1. Visionner la vidéo de nos experts, Dr D'Amours et Dre Ravitsky, sur la communication "professionnel de la santé - patient".

2. Visionner la vidéo du suivi de grossesse et consulter le document associé.

3. Compléter le questionnaire qui apparaît sous l'onglet *Évaluation*.

2. La rencontre professionnel de la santé - couple

Étapes de la prise de décision partagée
439,92 Ko, déposé le 18 déc. 2018

Versions audio des capsules vidéo

Formation : Prise de décision partagée pour le test de dépistage prénatal de la trisomie 21

4. La communication entre le professionnel et le patient

Activités | Évaluation | En savoir plus ... | Votre expert

Directives

Pour démontrer que vous avez bien atteint les objectifs visés par ce module, vous devez répondre à un questionnaire formatif en ligne. Pour y accéder, cliquez sur le lien ci-dessous.

Préparation à l'évaluation

- Ce questionnaire formatif porte sur l'ensemble du contenu couvert par le module 4 : *La communication: professionnel (le) - patient (e)* (vidéos), excluant le matériel sous l'onglet *En savoir plus...* .
- Avant de débuter le questionnaire, relisez les objectifs mentionnés sous l'onglet *Contenus* et assurez-vous que vous êtes en mesure de réaliser ce qui est attendu au terme de ce module.

Questionnaire du module 4 (À faire)
Disponible en tout temps
Tentatives : 0 / 1

© Université Laval, 2019. Tous droits réservés.

Formation : Prise de décision partagée pour le test de dépistage prénatal de la trisomie 21

4. La communication entre le professionnel et le patient

Activités Évaluation En savoir plus ... Votre expert

Télécharger les fichiers

Envie d'en savoir plus?

Voici des suggestions de ressources complémentaires en lien avec le thème de ce module. Bien que la consultation des ressources complémentaires soit facultative, ces ressources pourront vous permettre d'approfondir divers aspects liés au thème abordé au cours de ce module.

- Clarifier les valeurs et préférences des patients pour éclairer la prise de décision partagée sur le dépistage préventif

Clarifier les valeurs et préférences des patients pour éclairer la prise de décision partagée sur le dépistage préventif.pdf
178,73 Ko, déposé le 13 nov. 2018

© Université Laval, 2019. Tous droits réservés.

< Cours

Formation : Prise de décision partagée pour le test de dépistage prénatal de la trisomie 21

4. La communication entre le professionnel et le patient

Activités Évaluation En savoir plus ... Votre expert

Télécharger les fichiers

Invité spécial

Dr Denis D'Amours

Invité

M. Denis D'Amours

Clinicien
UMF de l'Hôpital Saint-François d'Assise

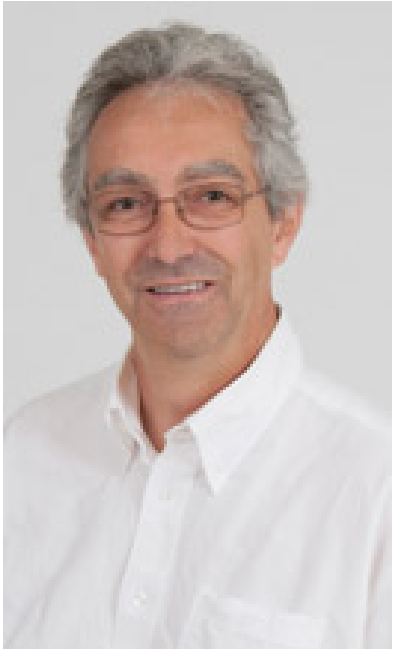

Vidéo de présentation

Dr D'Amours
7,72 Mo, déposé le 19 déc. 2018

Le docteur Denis D'Amours a obtenu sa certification du Collège des médecins de famille du Canada (CMFC) en 1983. Nommé Fellow du CMFC en 2001, il se joint à l'équipe de l'unité de médecine familiale (UMF) de l'Hôpital Saint-François-d 'Assise en 2002 et en devient le directeur en 2005.

Sa contribution à la vie de l'UMF est remarquable; il la défend ardemment en veillant également aux intérêts des unités d'hospitalisation en médecine familiale et des lits en soins palliatifs au sein du CHU de Québec. Le docteur D'Amours contribue également de façon exceptionnelle à la vie universitaire de l'Université Laval. D'ailleurs, sa contribution remarquable à la vie universitaire, hospitalière et groupe de médecin de famille lui permet de remporter le prix d'excellence du CMFC en 2014.

< Cours

Formation : Prise de décision partagée pour le test de dépistage prénatal de la trisomie 21

Simulation

Consignes

Cette simulation vidéo présente un exemple de consultation entre un professionnel de la santé et un couple lors de leur premier rendez-vous de suivi de grossesse. L'objectif est de. vous immerger dans une situation très près de la réalité vécue par les professionnels de la santé qui font le suivi de grossesse.. Par le biais de cette activité, vous serez amené à mettre en application ce que vous avez appris lors de la formation.

Bonne simulation!

Consultation en suivi de grossesse.

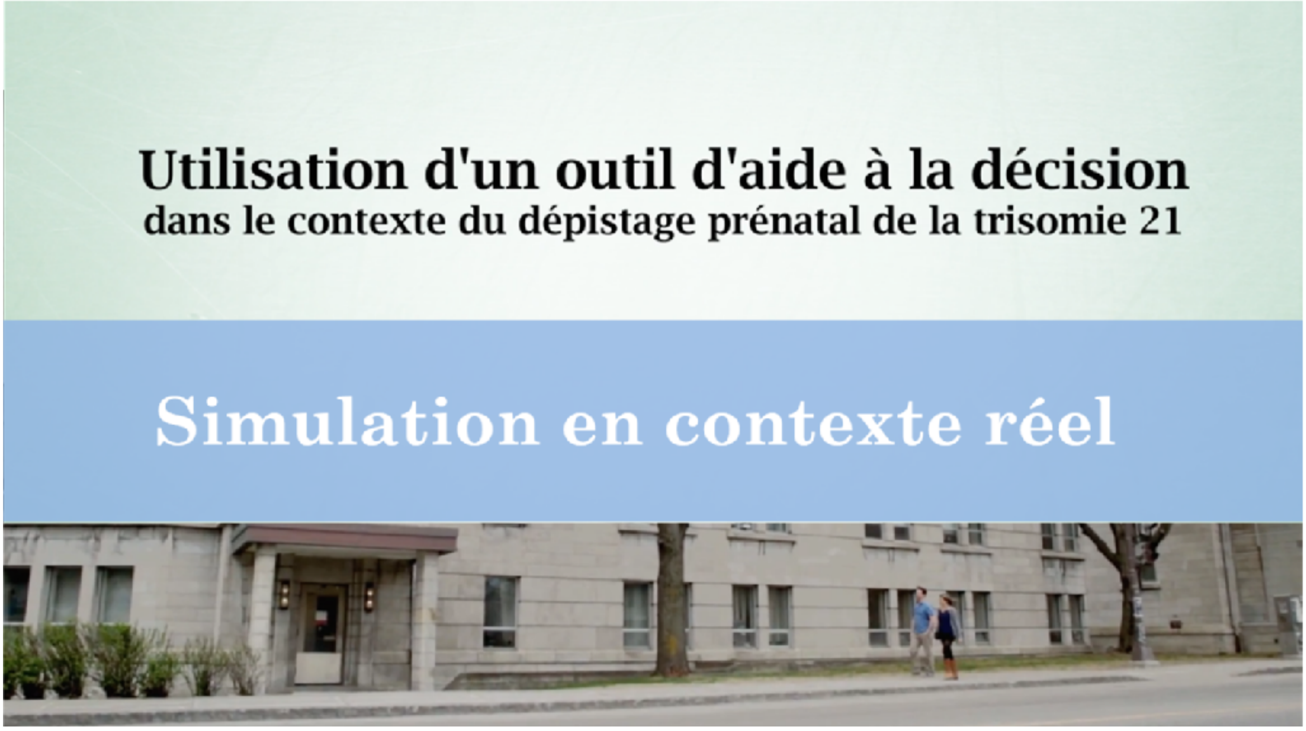

Accédez à la simulation

Attestation

Aucun contenu à afficher pour l'instant.

Formation : Prise de décision partagée pour le test de dépistage prénatal de la trisomie 21

Évaluation du programme

Télécharger les fichiers

Questions

Le questionnaire suivant vise à recueillir votre opinion sur cette formation en ligne afin d'améliorer et de mieux répondre aux besoins des différents professionnels de la santé lors des sessions futures. Vos suggestions et commentaires donneront des pistes essentielles à l'amélioration de l'approche pédagogique utilisée. Vos réponses à ce questionnaire sont strictement confidentielles. Seule la compilation statistique de toutes les réponses fournies et l'ensemble de vos commentaires sont transmis au responsable de la formation en ligne.

Questions d'évaluation du programme

Évaluation du programme
61,74 Ko, déposé le 29 sept. 2017

Évaluation de votre intention
439,81 Ko, déposé le 12 déc. 2018

© Université Laval, 2019. Tous droits réservés.

Formation : Prise de décision partagée pour le test de dépistage prénatal de la trisomie 21

Forum de discussion

Forum de discussion sur le contenu de la formation

Si vous souhaitez poser des questions sur le contenu de cette formation, exprimez-vous sur ce forum.

Forum de discussion sur divers sujets

Si vous souhaitez discuter de divers sujets, vous pouvez vous exprimer sur ce forum.

Espace libre : commentaires et suggestions

Si vous avez des commentaires et suggestions, exprimez-vous ici.

© Université Laval, 2019. Tous droits réservés.

Formation : Prise de décision partagée pour le test de dépistage prénatal de la trisomie 21

Ce site n'est pas encore publié. Il est présentement accessible uniquement à l'enseignant et aux assistants, s'il y a lieu.

Évaluations et résultats

Sommaire des résultats

> Introduction
> Liste des évaluations

Introduction

La formule d'évaluation se veut formative afin de faciliter et de suivre les apprentissages. Ainsi, une évaluation sous forme de questions à choix multiples et à correction automatisée est prévue à la fin de chaque module afin de valider les notions abordées.

Liste des évaluations

Les évaluations se trouvent dans la section "Outils", onglet "Questionnaires".

< Cours

Formation : Prise de décision partagée pour le test de dépistage prénatal de la trisomie 21

⚠ Ce site n'est pas encore publié. Il est présentement accessible uniquement à l'enseignant et aux assistants, s'il y a

Introduction

Plan de formation

Description de la formation

Contenu et activités

Évaluations et résultats

Contact

Outils

Envoi de courriel

Forums

Questionnaires

Contact

Si vous avez besoin de soutien pédagogique ou technique tout au long de votre formatio
les personnes-ressources dont les coordonnées apparaissent ci-dessous :

Coordonnées et disponibilités

Dre France Légaré (Responsable de la formation)
france.legare@fmed.ulaval.ca

Disponibilités
Pour toutes questions concernant le contenu de la formation, nous vous invitons à utilis

Soutien technique

Centre Techno-pedago (FM)
Pavillon Ferdinand-Vandry
www.fmed.ulaval.ca
techno-pedago@cifss.ulaval.ca

418 656-2131, poste 413057

Toutes sessions (du 1 janvier au 31 décembre)	
Lundi	8 h à 16 h 30
Mardi	8 h à 16 h 30
Mercredi	8 h à 16 h 30
Jeudi	8 h à 16 h 30
Vendredi	8 h à 16 h 30
