# Supplementary material for: Web-Based Training for Nurses on Using a Decision Aid to Support Shared Decision-making About Prenatal Screening: Parallel Controlled Trial
Source: JMIR Nurs. 2022 Jan 25;5(1):e31380. doi: 10.2196/31380 (PMC8826152; doi:10.2196/31380)
Supplement: Multimedia Appendix 4 [file nursing_v5i1e31380_app4.pdf]

Adaptation et évaluation de la formation en ligne sur la prise de décision partagée dans le contexte du dépistage prénatal de la trisomie 21 et des autres anomalies fœtales

Questionnaire 2 : Après la formation

Code site : ☐☐☐

Code patiente : ☐☐☐☐☐

Chaire de recherche du Canada sur la décision partagée et l'application des connaissances

Dre France Légaré, MD, PhD
Centre de recherche sur les soins et les services de première ligne de l'Université Laval (CERSSPL-UL), Département de médecine familiale et d'urgence, Faculté de médecine, Université Laval, Québec, Canada.

Version 03072019

Questionnaire

INTRO1 **POSER À TOUS**

Ce questionnaire vise à évaluer :

1) Votre intention d'utiliser un outil d'aide à la décision avec vos patients dans le contexte du dépistage prénatal,
2) Vos connaissances sur le contexte du dépistage prénatal et les pratiques l'entourant,
3) Votre degré de satisfaction par rapport à la formation,
4) L'acceptabilité d'une telle formation,
5) L'utilité perçue de la formation,
6) Vos réactions par rapport à la formation et,
7) Le niveau d'atteinte des objectifs de formation fixés.

Nous vous prions de répondre aux questions suivantes.

Pour chaque question, vous devez **cocher la case (<u>seulement une case</u>) qui correspond le mieux à votre réponse**.

Il n'y a pas de bonne ou de mauvaise réponse. C'est votre opinion qui nous intéresse.

Certains énoncés vous sembleront peut-être répétitifs. Ceci est dû à la méthodologie de l'étude. **Nous vous prions de répondre à <u>toutes</u> les questions.**

Question générale

Q1-POSER À TOUS [mention simple – en ordre]

Avez-vous complété l'ensemble de la formation ? La formation est considérée comme complétée lorsque l'ensemble des modules ont été consultés.

a) Oui, j'ai complété l'ensemble de la formation.
b) Non, je n'ai pas complété l'ensemble des modules.
c) Autre. Veuillez inscrire votre commentaire :
____

1) Intention

Suite à la formation, nous aimerions connaitre votre intention d'utiliser un outil d'aide à la décision avec vos patients confrontés à la décision en lien avec le dépistage prénatal. Veuillez s'il vous plaît répondre à chacune des questions suivantes en indiquant le numéro qui correspond le mieux à votre opinion par rapport au comportement indiqué. Certaines questions peuvent vous sembler similaires, mais elles mesurent des aspects différents du comportement énoncé.

> *Note : Les outils d'aide à la décision aident les patients et les professionnels de la santé dans leur prise de décision en rendant explicite la décision à prendre, en fournissant de l'information au sujet des options et des résultats sur la santé et en clarifiant les valeurs personnelles. Il est important de noter que les outils d'aide à la décision ont été conçus afin d'accompagner, et non de remplacer, les conseils d'un professionnel de la santé.*
>
> *Institut de Recherche, Hôpital d'Ottawa*

		**Fortement en désaccord**						**Fortement en accord**
1.	J'ai l'intention d'utiliser un outil d'aide à la décision dans le contexte du dépistage prénatal	☐	☐	☐	☐	☐	☐	☐

		**0-20%**	**21-40%**	**41-60%**	**61-80%**	**81-100%**
2.	Au meilleur de ma connaissance, la proportion de mes collègues qui utilisent un outil d'aide à la décision dans le contexte du dépistage prénatal est de:	☐	☐	☐	☐	☐

		Totalement en désaccord						Totalement en accord
3.	Je suis confiant(e) que je pourrais utiliser un outil d'aide à la décision dans le contexte du dépistage prénatal si je le voulais	☐	☐	☐	☐	☐	☐	☐
		**Totalement en désaccord**						**Totalement en accord**
4.	Il est éthique d'utiliser un outil d'aide à la décision dans le contexte du dépistage prénatal	☐	☐	☐	☐	☐	☐	☐
		**Extrêmement difficile**						**Extrêmement facile**
5.	Pour moi, utiliser un outil d'aide à la décision dans le contexte du dépistage prénatal serait :	☐	☐	☐	☐	☐	☐	☐
		**Jamais**						**Toujours**
6.	Pensez maintenant à un collègue de travail que vous respectez comme professionnel. À votre avis, utilise-t-il un outil d'aide à la décision dans le contexte du dépistage prénatal ?	☐	☐	☐	☐	☐	☐	☐
		**Fortement en désaccord**						**Fortement en accord**
7.	Je planifie d'utiliser un outil d'aide à la décision dans le contexte du dépistage prénatal	☐	☐	☐	☐	☐	☐	☐

		**Inutile**						**Utile**
8.	De façon générale, je pense qu'utiliser un outil d'aide à la décision dans le contexte du dépistage prénatal serait pour moi :	☐	☐	☐	☐	☐	☐	☐
		**Fortement en désaccord**						**Fortement en accord**
9.	La plupart des personnes importantes pour moi dans ma profession utilisent un outil d'aide à la décision dans le contexte du dépistage prénatal	☐	☐	☐	☐	☐	☐	☐
		**Totalement en désaccord**						**Totalement en accord**
10.	Il est acceptable d'utiliser un outil d'aide à la décision dans le contexte du dépistage prénatal	☐	☐	☐	☐	☐	☐	☐
		**Fortement en désaccord**						**Fortement en accord**
11.	Je suis capable d'utiliser un outil d'aide à la décision dans le contexte du dépistage prénatal	☐	☐	☐	☐	☐	☐	☐
		**Néfaste**						**Bénéfique**
12.	De façon générale, je pense qu'utiliser un outil d'aide à la décision dans le contexte du dépistage prénatal serait pour moi:	☐	☐	☐	☐	☐	☐	☐

Légaré et al. 2014

2) Connaissances

TEXTE1 POSER À TOUS

Vous trouverez dans cette section 20 questions à choix multiples. Elles visent à mesurer vos connaissances sur le dépistage prénatal et les pratiques qui l'entourent :

Veuillez svp, cocher la bonne réponse (une case seulement).

Q2-POSER À TOUS [mention simple – en ordre]

Voici quelques énoncés concernant la trisomie 21. Cochez l'énoncé qui est **faux**.

a) La trisomie 21 est causée par la présence d'un 3e chromosome à la 21e paire
b) La trisomie 21 entraîne une déficience intellectuelle pouvant varier de légère à modérée, parfois grave
c) La majorité des cas de trisomie 21 sont de type héréditaire
d) La trisomie 21 peut avoir certaines de ces caractéristiques : faible tonus musculaire, grande souplesse des articulations, un risque plus grand d'avoir des problèmes de vue ou des malformations cardiaques et/ou des problèmes gastro-intestinaux
e) Le risque de trisomie 21 augmente avec l'âge maternel

Q3-POSER À TOUS [mention simple – en ordre]

Voici différents énoncés par rapport au test biochimique intégré. Cochez l'énoncé qui est **vrai**.

a) La première prise de sang pour le test biochimique intégré se fait la entre la 9ème et la 13ème semaine de grossesse
b) Le délai d'attente des résultats pour le test biochimique intégré, le test biochimique intégré et la clarté nucale est d'environ 2-3 jours.
c) Le taux de détection du test biochimique intégré seul est de 85%.
d) Le test biochimique intégré est indiqué pour les grossesses multiples.

Q4-POSER À TOUS [mention simple – en ordre]

Comment peut-on bonifier la fiabilité du test biochimique intégré? Cochez l'énoncé qui est **vrai**.

a) En intégrant la mesure de la clarté nucale
b) En intégrant une prise de sang au 3e trimestre
c) En faisant les prélèvements tôt le matin
d) En prenant compte de la date des dernières menstruations (DDM)

Q5-POSER À TOUS [mention simple – en ordre]

Voici quelques affirmations concernant le test biochimique intégré. Cochez l'énoncé qui est **faux**.

a) Le seuil du risque pour ce test qui a été retenu est de 1/300 pour une grossesse à terme.
b) Le taux de faux positif est d'environ 3%.
c) L'âge maternel est exclu du calcul du risque.
d) Le dépistage biochimique intégré comprend les marqueurs biochimiques au 1er et 2e trimestre.

Q6-POSER À TOUS [mention simple – en ordre]

Vrai ou faux. Le degré de stimulation et le soutien offerts aux enfants trisomiques ont une influence sur les limites intellectuelles que présentera l'enfant avec la trisomie 21. Les enfants trisomiques peuvent également être scolarisés dans des écoles traditionnelles.

a) Vrai
b) Faux

Q7-POSER À TOUS [mention simple – en ordre]

Voici quelques énoncés concernant l'amniocentèse. Cochez l'énoncé qui est **faux**.

a) L'amniocentèse est un test diagnostique et apporte une certitude si l'enfant porte ou non la trisomie 21
b) L'amniocentèse consiste à prélever du liquide amniotique dans le ventre de la femme enceinte à l'aide d'une fine aiguille.
c) Les complications possibles de l'amniocentèse peuvent être une perte légère de liquide amniotique, des contractions utérines, des douleurs abdominales et une fausse couche.
d) L'amniocentèse peut fournir des renseignements précis sur le degré de déficience intellectuelle, sur la présence de malformations physiques ou sur d'éventuels problèmes de santé.
e) Les résultats d'amniocentèse peuvent causer de l'anxiété par rapport à la nécessité de prendre une décision concernant la poursuite ou l'interruption de grossesse.

Q8-POSER À TOUS [mention simple – en ordre]

Quels sont les enjeux éthiques liés au dépistage prénatal? Choisissez la combinaison appropriée parmi les options ci-dessous.

1. Routinisation de l'offre du test de dépistage
2. Décision de faire ou ne pas faire le test diagnostique
3. Impact sociétal des décisions individuelles

a) 1. et 2.
b) 1. et 3.

c) 2. et 3.
d) Toutes ces réponses.
e) Aucune de ces réponses.

Q9-POSER À TOUS [mention simple – en ordre]
Vrai ou faux. Plus la femme est informée au sujet du dépistage prénatal, plus elle sera davantage portée à refuser le test.

a) Vrai
b) Faux

Q10-POSER À TOUS [mention simple – en ordre]
Voici quelques actions à entreprendre pour tenir compte des enjeux éthiques liés au dépistage prénatal. Cochez l'énoncé qui est FAUX.

a) Offrir de l'information relative aux tests de dépistage de la trisomie 21 à toutes les femmes, et ce, gratuitement.
b) Éviter la standardisation de l'information véhiculée dans le cadre du dépistage prénatal.
c) Obtenir des résultats fiables des tests afin de donner un réel choix aux parents. Leur rendement doit répondre aux normes cliniques actuelles, par ailleurs évolutives.
d) Informer les parents, pour qu'ils puissent faire un choix libre et éclairé, de ; les aspects médicaux des tests, l'évaluation des risques, l'expérience de la vie avec la trisomie 21, l'avortement et le deuil.

Q11-POSER À TOUS [mention simple – en ordre]
Voici quelques énoncés par rapport au consentement libre et éclairé au dépistage. Choisissez la combinaison appropriée parmi les options ci-dessous.

1. Le test de dépistage doit être offert sur une base volontaire et non prescrit de manière systématique.
2. Toute l'information nécessaire à la prise de décision doit être transmise au préalable
3. Les professionnels doivent transmettre l'information d'une manière non-directive
4. Les femmes enceintes les couples doivent avoir la possibilité de réfléchir avant de prendre leur décision.

a) 1. 2. 3.
b) 1. 3. 4.
c) 2. 3. 4
d) Aucune de ces réponses
e) Toutes ces réponses

Q12-POSER À TOUS [mention simple – en ordre]
Sur quoi repose le processus de décision partagée? Choisissez la combinaison appropriée parmi les options ci-dessous.

1. Les données probantes
2. Les valeurs et les préférences des patients
3. Les risques et les bénéfices de toutes les options disponibles, excluant celle de ne rien faire
4. Les risques et les bénéfices de toutes les options disponibles, y compris celle de ne rien faire

a) 1-2
b) 1-2-3
c) 1-2-4
d) 1-3
e) 2-4

Q13-POSER À TOUS [mention simple – en ordre]
Qui sont les personnes impliquées dans le processus de décision partagée? Cochez la bonne réponse.

a) Les professionnels de la santé et les patients
b) Les professionnels de la santé, les patients et parfois la famille
c) Les professionnels de la santé
d) Les patients
e) Les patients et parfois leur famille

Q14-POSER À TOUS [mention simple – en ordre]
Cochez la meilleure combinaison d'énoncés concernant le modèle de prise de décision partagée.

1. Le professionnel de la santé tient un rôle passif
2. Le professionnel de la santé tient un rôle actif
3. Le patient tient un rôle passif
4. Le patient tient un rôle actif
5. Le médecin présente l'information
6. Le médecin présente les options disponibles
7. Le patient communique ses préférences
8. La prise de décision partagée nécessite de la négociation et un consensus

a) 1. 4. 5. 6. 7.
b) 2. 3. 5. 6. 7.
c) 1. 4. 5. 7. 8

d) 2. 4. 5. 6. 7.
e) 2. 4. 5. 7. 8.

Q15-POSER À TOUS [mention simple – en ordre]
Voici quelques impacts de la prise de décision partagée. Cochez l'énoncé qui est **faux**.

a) Amélioration du confort décisionnel chez le patient
b) Amélioration de la qualité de la rencontre clinique en augmentant sa durée
c) Concrétisation des attentes des patients.
d) Bonification des connaissances du patient au niveau des bénéfices, des risques et de l'incertitude scientifique
e) Amélioration du confort décisionnel du professionnel de la santé et celui du patient

Q16-POSER À TOUS [mention simple – en ordre]
Dans quel contexte il est requis d'avoir recours à la décision partagée? Cochez l'énoncé qui est **vrai.**

a) Lorsqu'il y a une multitude d'options
b) Lorsque le patient est inapte à prendre une décision
c) Lorsqu'il y a un conflit d'intérêt et/ou de valeurs entre le professionnel et le patient.
d) Lorsqu'il y a un consensus dans la littérature et la pratique sur la meilleure option offerte pour tous.

Q17-POSER À TOUS [mention simple – en ordre]
Choisissez la séquence chronologique qui représente les étapes de la prise de décision partagée.

1. Discuter des avantages/risques potentiels (nature probabiliste des données probantes)
2. Présenter les recommandations
3. Explorer l'efficacité personnelle du patient
4. Définir le problème/identifier la décision à prendre
5. Vérifier la compréhension
6. Identifier les valeurs/préférences du patient
7. Prendre/différer la décision et prendre les dispositions nécessaires pour le suivi
8. Présenter les options

a) 8. 1. 6. 3. 5. 2. 4. 7.
b) 3. 2. 1. 6. 8. 4. 5. 7.
c) 6. 3. 4. 8. 1. 2. 5. 7.
d) 4. 8. 1. 6. 3. 2. 5. 7.

Q18-POSER À TOUS [mention simple – en ordre]

Voici quelques énoncés sur les outils d'aide à la décision. Cochez la combinaison d'énoncés qui est **vraie**.

1. Les outils d'aide à la décision peuvent supporter les patients dans leur processus décisionnel.
2. Ils sont utilisés pour bonifier, et non remplacer, l'interaction entre le clinicien et le patient.
3. Ils peuvent être utilisés avant la rencontre avec le clinicien (pour se préparer) ou lors de la rencontre pour faciliter la prise de décision.
4. Ils fournissent de l'information à propos des options disponibles et leurs conséquences.

a) 1. 2. 3.
b) 1. 3. 4.
c) 2. 3. 4.
d) Toutes ces réponses
e) Aucune de ces réponses

Q19-POSER À TOUS [mention simple – en ordre]
Vous recevez le rapport du dépistage de Mme Tremblay. Celle-ci a 33 ans et a réalisé le test biochimique intégré et la clarté nucale. Son risque calculé est de 1/200.

Voici quelques énoncés concernant la signification du faux positif (considérant qu'il est de 3%). Choisissez l'énoncé qui est **vrai**.

a) Son enfant a 3% de chance d'être porteur de la trisomie 21.
b) Son enfant a 97% de chance d'être porteur de la trisomie 21.
c) 3% des femmes testées auront été inquiétées à tort (risque élevé, fœtus non porteur)
d) 97 % des femmes testées auront été inquiétées à tort (risque élevé, fœtus non porteur)
e) Son enfant a 0,015% de chances d'être trisomique (risque [1/200] x faux positif [3%])

Q20-POSER À TOUS [mention simple – en ordre]
Vous recevez le rapport du dépistage de Mme Dubois. Celle-ci a 27 ans et a réalisé ses deux prises de sang et a eu sa clarté nucale. Son risque calculé est de <1/10 000.

Voici une liste d'éléments à discuter avec la patiente concernant son résultat. Choisissez l'énoncé qui est **vrai.**

a) Le risque est considéré assez faible pour ne pas considérer l'amniocentèse.
b) Il n'y a pas de possibilité d'avoir un fœtus atteint de la trisomie 21.
c) Le test effectué est un outil fiable pour détecter tous les types d'anomalies congénitales.
d) Il n'est pas nécessaire d'expliquer la notion de risque faible à la patiente compte tenu de la normalité du résultat.

Q21-POSER À TOUS [mention simple – en ordre]

Vous recevez le rapport du dépistage de Mme Lévesque. Celle-ci a 31 ans et a réalisé ses deux prises de sang et a eu sa clarté nucale. Son risque calculé est de 1/210.

Voici une liste d'éléments à discuter avec la patiente concernant son résultat. Choisissez l'énoncé qui est **faux**.

a) Il y a 5% de faux positif à ce dépistage.
b) Le risque est considéré assez faible pour ne pas considérer l'amniocentèse.
c) Le risque calculé selon l'âge maternel et les marqueurs biochimiques est plus élevé que le seuil de dépistage.
d) Le test effectué est un dépistage et comporte ses limites.

3) Satisfaction

TEXT2 POSER À TOUS

Nous aimerions connaitre votre niveau de satisfaction par rapport au contenu, aux formateurs et à l'ensemble de la formation. Veuillez répondre «fortement en désaccord», «plutôt en désaccord», «neutre», «plutôt en accord», ou «fortement en accord» à chacun des énoncés suivants en cochant la réponse voulue.

Q22A-E-POSER À TOUS [mention simple – en rotation]

Satisfaction par rapport contenu de la formation	Fortement en désaccord	Plutôt en désaccord	Ni en désaccord, ni en accord	Plutôt en accord	Fortement en accord
a) Le contenu de la formation a contribué à l'amélioration de mes connaissances	☐	☐	☐	☐	☐
b) Le contenu de la formation était nouveau pour moi	☐	☐	☐	☐	☐
c) Le contenu de la formation était pertinent pour la pratique clinique	☐	☐	☐	☐	☐
d) Le contenu de la formation correspond aux objectifs de formation présentés	☐	☐	☐	☐	☐
e) La formation a été didactiquement bien mise en œuvre	☐	☐	☐	☐	☐

Schmidt, 2007.

Q23A-F POSER À TOUS [mention simple – en rotation]

Satisfaction par rapport aux intervenants à l'ensemble de la formation	Fortement en désaccord	Plutôt en désaccord	Ni en désaccord, ni en accord	Plutôt en accord	Fortement en accord
a) Dans l'ensemble, j'ai bien aimé la formation	☐	☐	☐	☐	☐
b) Dans l'ensemble, la formation était bien organisée	☐	☐	☐	☐	☐
c) Le contenu de la formation a été présenté de manière compréhensible	☐	☐	☐	☐	☐
d) La formation avait une structure claire	☐	☐	☐	☐	☐
e) La durée de formation était appropriée au temps alloué pour la formation	☐	☐	☐	☐	☐
f) Les formateurs étaient bien préparés et organisés	☐	☐	☐	☐	☐

4) Acceptabilité

TEXT3 POSER À TOUS

Nous aimerions connaitre à quel point vous trouvez cette formation acceptable. Veuillez répondre «fortement en désaccord», «plutôt en désaccord», «neutre», «plutôt en accord», ou «fortement en accord» à chacun des énoncés suivants en cochant la réponse voulue.

Q24A-E-POSER À TOUS [mention simple – en rotation]

4) Acceptabilité de la formation	Fortement en désaccord	Plutôt en désaccord	Ni en désaccord, ni en accord	Plutôt en accord	Fortement en accord
a) La quantité d'information et de matériel présenté contenue dans cette formation est acceptable	☐	☐	☐	☐	☐
b) Le format d'auto-formation en ligne utilisé est acceptable	☐	☐	☐	☐	☐
c) La formation sur la prise de décision partagée dans le contexte du dépistage prénatal est acceptable	☐	☐	☐	☐	☐
d) Le nombre de crédits alloués pour la formation est acceptable	☐	☐	☐	☐	☐
e) Je recommanderais cette formation à un collègue	☐	☐	☐	☐	☐

Kasper & al, 2017

5) Utilité perçue

TEXT4 POSER À TOUS

Nous aimerions connaitre votre perception par rapport à l'utilité de cette formation. Veuillez répondre «fortement en désaccord», «plutôt en désaccord», «neutre», «plutôt en accord», ou «fortement en accord» à chacun des énoncés suivants en cochant la réponse voulue.

Giangreco, Sebastiano & Peccei, 2009.

Q25A-E-POSER À TOUS [mention simple – en rotation]

5) Utilité perçue de la formation	Fortement en désaccord	Plutôt en désaccord	Ni en désaccord, ni en accord	Plutôt en accord	Fortement en accord
a) Les sujets abordés sont utiles pour mes responsabilités professionnelles	☐	☐	☐	☐	☐
b) Les sujets abordés sont pertinents pour le développement de ma carrière	☐	☐	☐	☐	☐
c) Les sujets abordés sont pertinents par rapport à mes besoins d'apprentissage personnels	☐	☐	☐	☐	☐
d) Les sujets abordés sont cohérents avec les objectifs déclarés	☐	☐	☐	☐	☐
e) Il y a un bon équilibre entre la théorie et la pratique	☐	☐	☐	☐	☐

Giangreco, Sebastiano & Peccei, 2009.

6) Réactions

TEXT5 POSER À TOUS

Nous aimerions connaitre vos réactions à l'issue de la formation. Veuillez répondre «fortement en désaccord», «plutôt en désaccord», «neutre», «plutôt en accord», ou «fortement en accord» à chacun des énoncés suivants en cochant la réponse voulue.

Kirkpatrick & Kirkpatrick, 2009

Q26A-K-POSER À TOUS [mention simple – en rotation]

6) Aspect pédagogique	Fortement en désaccord	Plutôt en désaccord	Ni en désaccord, ni en accord	Plutôt en accord	Fortement en accord
a) Le contenu de l'activité était pertinent quant à ma pratique	☐	☐	☐	☐	☐
b) Les objectifs énoncés ont été atteints	☐	☐	☐	☐	☐
c) Le conférencier a présenté l'information de façon claire et efficace	☐	☐	☐	☐	☐
d) La documentation fournie me sera utile	☐	☐	☐	☐	☐
e) Le temps alloué (3h) pour la formation était suffisant	☐	☐	☐	☐	☐
h) L'organisation générale de l'activité était excellente (programme, audiovisuel, animation, simulation).	☐	☐	☐	☐	☐
i) Cette formation aura un impact sur ma pratique	☐	☐	☐	☐	☐
j) Absence de biais commerciaux	☐	☐	☐	☐	☐
k) Respecte le Code d'éthique des intervenants en éducation médicale continue	☐	☐	☐	☐	☐

Kirkpatrick & Kirkpatrick, 2009

7) Niveau d'atteinte des objectifs

TEXT5 POSER À TOUS

Nous aimerions connaitre à quel point vous avez atteint les objectifs de formation fixés. Veuillez répondre «fortement en désaccord», «plutôt en désaccord», «neutre», «plutôt en accord», ou «fortement en accord» à chacun des énoncés suivants en cochant la réponse voulue.

Q27A-K-POSER À TOUS [mention simple – en rotation]

7) À l'issue de cette formation, je suis en mesure de :	Fortement en désaccord	Plutôt en désaccord	Ni en désaccord, ni en accord	Plutôt en accord	Fortement en accord
a) Définir le concept de décision partagée	☐	☐	☐	☐	☐
b) Reconnaître la pertinence de la décision partagée	☐	☐	☐	☐	☐
c) Identifier les éléments favorisant ou entravant l'implantation de la décision partagée dans les milieux cliniques	☐	☐	☐	☐	☐
d) Énumérer les avantages pour les cliniciens (nes) et les patients (es)	☐	☐	☐	☐	☐
e) Nommer les objectifs du dépistage prénatal de la trisomie 21	☐	☐	☐	☐	☐
f) Identifier les points de décision dans le contexte québécois du dépistage prénatal de la trisomie 21	☐	☐	☐	☐	☐
g) Identifier les enjeux éthiques et sociaux du dépistage prénatal	☐	☐	☐	☐	☐
h) Identifier les points de décision dans le contexte du dépistage prénatal de la trisomie 21	☐	☐	☐	☐	☐
i) Identifier les principales caractéristiques qui distinguent un outil d'aide à la décision d'un dépliant d'information	☐	☐	☐	☐	☐
j) Appliquer des stratégies efficaces afin de communiquer les options associées au dépistage prénatal de la trisomie 21 ainsi que les avantages et inconvénients qui y sont reliés	☐	☐	☐	☐	☐
k) Utiliser des stratégies efficaces afin d'identifier les valeurs et les préférences des patientes dans le contexte du dépistage prénatal de la trisomie 21	☐	☐	☐	☐	☐

Q28-POSER À TOUS [question ouverte]

8) Commentaires généraux

*** Nous vous remercions d'avoir complété ce questionnaire ***
